# Comparative genomics and physiological investigation supported safety, cold adaptation, efficient hydrolytic and plant growth-promoting potential of psychrotrophic *Glutamicibacter arilaitensis* LJH19, isolated from night-soil compost

**DOI:** 10.1186/s12864-021-07632-z

**Published:** 2021-04-28

**Authors:** Shruti Sinai Borker, Aman Thakur, Sanjeet Kumar, Sareeka Kumari, Rakshak Kumar, Sanjay Kumar

**Affiliations:** 1 Biotechnology Division, CSIR-Institute of Himalayan Bioresource Technology Palampur, Palampur, Himachal Pradesh 176061 India; 2 Academy of Scientific and Innovative Research (AcSIR), CSIR- Human Resource Development Centre, Ghaziabad, Uttar Pradesh 201 002 India

**Keywords:** Winter dry toilet, Polysaccharide metabolism, Indole acetic acid, siderophore, type III PKS

## Abstract

**Background:**

Night-soil compost (NSC) has traditionally been conserving water and a source of organic manure in northwestern Himalaya. Lately, this traditional method is declining due to modernization, its unhygienic conditions, and social apprehensions. Reduction in the age-old traditional practice has led to excessive chemical fertilizers and water shortage in the eco-sensitive region. In the current study, a bacterium has been analyzed for its safety, cold-adaptation, efficient degradation, and plant growth-promoting (PGP) attributes for its possible application as a safe bioinoculant in psychrotrophic bacterial consortia for improved night-soil composting.

**Results:**

*Glutamicibacter arilaitensis* LJH19, a psychrotrophic bacterium, was isolated from the NSC of Lahaul valley in northwestern Himalaya. The strain exhibited amylase (186.76 ± 19.28 U/mg), cellulase (21.85 ± 0.7 U/mg), and xylanase (11.31 ± 0.51 U/mg) activities at 10 °C. Possessing efficient hydrolytic activities at low-temperature garners the capability of efficient composting to LJH19. Additionally, the strain possessed multiple PGP traits such as indole acetic acid production (166.11 ± 5.7 μg/ml), siderophore production (85.72 ± 1.06% psu), and phosphate solubilization (44.76 ± 1.5 μg/ml). Enhanced germination index and germination rate of pea seeds under the LJH19 inoculation further supported the bacterium’s PGP potential. Whole-genome sequencing (3,602,821 bps) and genome mining endorsed the cold adaptation, degradation of polysaccharides, and PGP traits of LJH19. Biosynthetic gene clusters for type III polyketide synthase (PKS), terpene, and siderophore supplemented the endorsement of LJH19 as a potential PGP bacterium. Comparative genomics within the genus revealed 217 unique genes specific to hydrolytic and PGP activity.

**Conclusion:**

The physiological and genomic evidence promotes LJH19 as a potentially safe bio-inoculant to formulate psychrotrophic bacterial consortia for accelerated degradation and improved night-soil compost.

**Supplementary Information:**

The online version contains supplementary material available at 10.1186/s12864-021-07632-z.

## Background

**Fig. 1:**
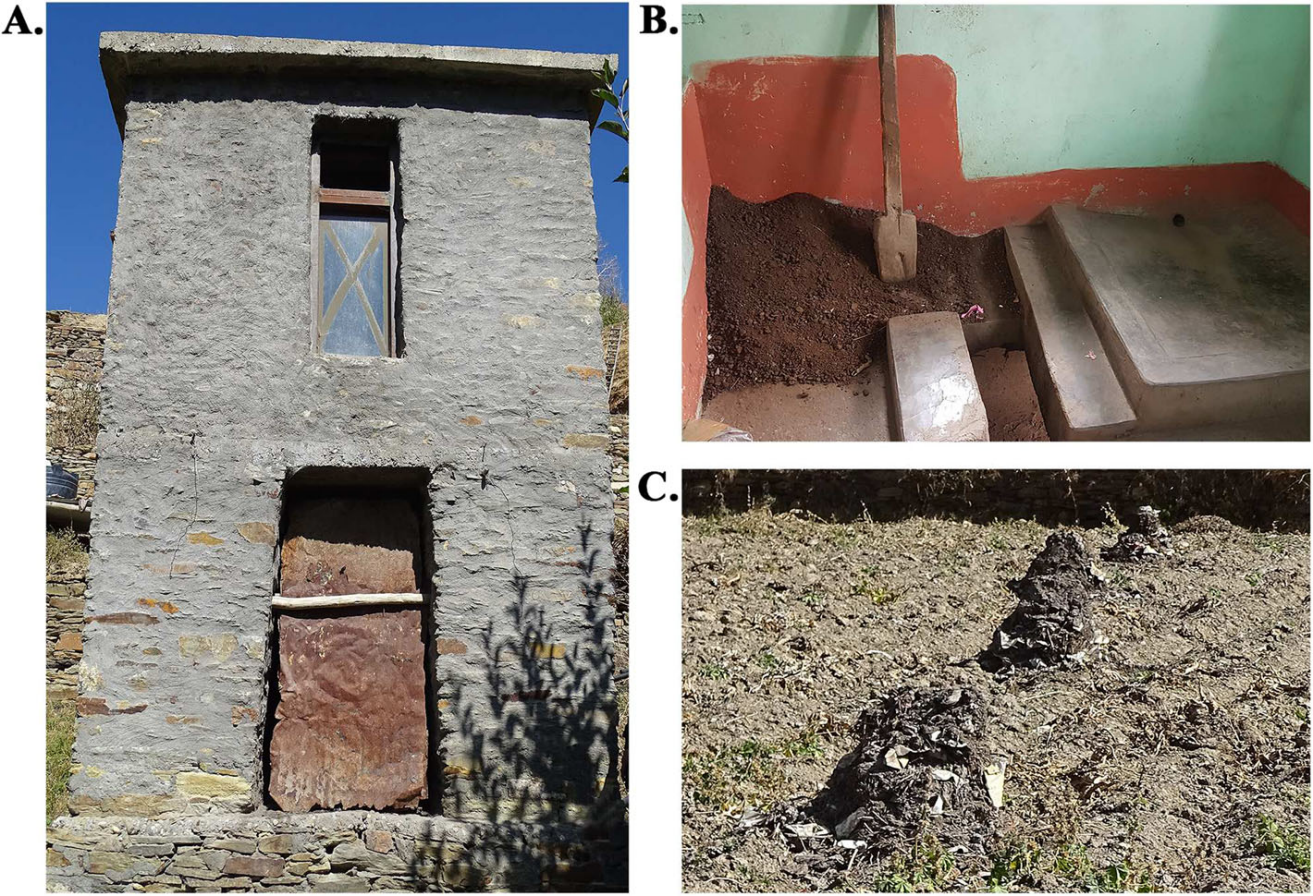
Traditional winter dry toilet of Lahaul valley. **a** Traditional winter dry toilet attached to the living room of the main house. The dry toilet structure is made up of two-storey construction, i.e., defaecation room and collection chamber. **b** Inside view of the defaecation room. After defaecation, the night-soil is thoroughly covered with mixture locally referred as ‘*fot*’ (composed of dry cattle/sheep dung, kitchen ash, and dry grass/leaves). **c** NSC pile dumped in open fields for further curing

The highland agro system of the northwestern Himalaya lacks productivity and soil fertility due to extreme weather conditions like heavy snowfall, avalanches, landslides, soil erosion, and scanty rainfall [1]. To meet the high demand for manure and water shortage during winter, the traditional method of composting human excreta (night-soil) using dry toilets is prevalent in this region [2–4]. The dry toilet consists of a defaecation room (upper storey) and a collection chamber (lower storey) (Fig. 1a). After the defaecation, the feces is covered with ‘*fot*’ composed of dry mixtures of dry cattle/sheep dung, kitchen ash, sand, dry grass/leaves, etc. (Fig. 1b). The night-soil is decomposed with time and dumped into the open fields in a series of piles for further curing (Fig. 1c). Lately, the night-soil composting practice is declining, promoting excessive chemical fertilizers use in ecologically vulnerable high altitude farmlands [3]. Promotion of the safe and hygienic winter dry toilets aided with scientific intervention is necessary to sustain the agro-ecosystem and conservation of water in such highland areas.

The foul odor of the winter dry toilet has been one of the main reasons for the decline of this practice. In a cold climate, the lower microbial load in the initial composting process delays the composting process due to slower biomass degradation, and psychrotrophic bacteria play a crucial role in low-temperature composting [5]. Plant growth-promoting (PGP) bacteria play an important role in maintaining soil fertility by increasing the availability of the nutrients, such as iron, nitrogen, phosphorous, and by producing phytohormones (indole acetic acid- IAA), growth regulators (siderophores), and solubilizing phosphate to modulate plant growth and development [6, 7]. In isolating the psychrotrophic, efficiently degrading strains with PGP potential to formulate psychrotrophic bacterial consortium for application in night-soil composting, we obtained a bacterial strain LJH19 with remarkable PGP traits and efficient hydrolytic activity in in-vitro assays. Owing to its cold adaptation, efficient hydrolytic activity, PGP potential, and origin from fecal compost, whole-genome sequencing was performed to elucidate the genetic basis of the catabolic activities, PGP traits, and analysis for pathogenicity determinants.

Further, to explore the habitat-specific gene repertoire, we performed comparative genomics of LJH19 with all the available strains of the same genus. A comparison was withdrawn with the closely related strains based on a unique genome region across the strain LJH19. Biosynthetic gene clusters in the genome of LJH19 were also identified, and further to evaluate bacterial safety, the presence of antibiotic resistance gene cluster across all the strains was assessed. It is important to study each potential bacterium individually to formulate an efficient psychrotrophic bacterial consortium. The current study aims to establish strain LJH19 as a potential bio-inoculant for application in consortia for night-soil composting.

## Results and discussion

### Physico-chemical properties of night-soil compost (NSC) samples and bacterial characterization

The compost samples were collected from the compost pile randomly in triplicate from the collection chamber of the traditional dry toilet ‘*ghop*’ (Fig. 1a). The compost sample was obtained from the core of the pile; the temperature, pH, and electrical conductivity (EC) of the collected samples were 9.9 °C, 10, and 1674 μS, respectively. The available nitrogen, phosphorous, and potassium in the collected NSC samples were 2297.6 ± 99.4 ppm, 117.11 ± 0.34 ppm, and 22,534.11 ± 73.08 ppm, respectively.

In an attempt to explore the bacterial diversity from NSC, 130 bacterial strains belonging to varied taxonomic genera were obtained based on their hydrolytic activities in different substrates and PGP traits (unpublished data). One such efficient hydrolytic bacterial colony was an opaque, yellow-pigmented bacterium LJH19 that showed multiple hydrolytic activities. The bacterium could survive at varying temperatures (4–37 °C) and showed optimum growth at 10 °C, pH 7 (Table 1). The bacterium showed hydrolytic activity against substrates like corn starch, CMC, and birchwood xylan on plate-based assays at a varied temperature of 4–37 °C, and the most efficient activity was obtained at 10 °C (Table 1, Supplementary Figure S1). Gene sequence similarity based on partial 16S rRNA gene (NCBI accession no. MT349443) related the bacteria to *G. arilaitensis* Re117^T^ with 100% identity and coverage of 96.5% in EzTaxon Biocloud (https://www.ezbiocloud.net/identify). Quantitatively, LJH19 showed enzyme activity at varying temperatures (4, 10, 15, 20, 28, and 37 °C), and the best production was obtained at 10 °C. At 10 °C, the strain LJH19 exhibited production of amylase enzyme with a specific activity of 186.76 ± 19.28 U/mg (Supplementary Figure S2) using corn starch as substrate, cellulase enzyme with a specific activity of 21.85 ± 0.7 U/mg (Supplementary Figure S2) using CMC as a substrate and xylanase enzyme with a specific activity of 11.31 ± 0.51 U/mg (Supplementary Figure S2) using birchwood xylan as a substrate. It has been hypothesized that psychrotrophic bacteria play a crucial role in low-temperature composting, and it is critical for a bacterium to possess enzymatic activities to ensure efficient composting [5, 8, 9]. Like LJH19, other strains of genus *Glutamicibacter* have also been reported to possess hydrolytic enzymes such as amylase and cellulase [10, 11]. *Glutamicibacter* strains have been reported from varied niche areas [12], and its reclassification originates from a much diverse genera *Arthrobacter* [13]. Genus *Arthrobacter* has also been reported from a harsh cold environment with potential hydrolytic enzymes [14, 15]. With survival at a temperature as low as 4 °C and efficient hydrolytic activity against complex polysaccharides (starch, cellulose, and xylan), the strain LJH19 was chosen as a potential candidate for psychrotrophic consortia for accelerated degradation of NSC at low ambient temperature.

**Table 1:** Physiological characterization, hydrolytic, plant growth promoting, and pathogenic attributes of *G*. *arilaitensis* LJH19

Characteristic	***G. arilaitensis*** LJH19
Source	Night-soil compost
Growth condition ^a^	
Temperature range	4–37 °C (10 °C)
pH range	7–11 (7)
NaCl range	1–9% (1%)
Hydrolysis on agar plates ^b^	
Corn starch	+ ve (15)
Carboxymethylcellulose (CMC)	+ ve (5.66)
Birchwood xylan	+ ve (2.8)
Tributyrin	+ ve (1.8)
Enzyme assays	
Amylase	186.76 ± 19.28 U/mg
Cellulase	21.85 ± 0.7 U/mg
Xylanase	11.31 ± 0.51 U/mg
PGP trait	
IAA production	166.11 ± 5.7 μg/ml
Siderophore production^c^	85.72 ± 1.06% siderophore unit (1.5)
Phosphate solubilisation^d^	44.76 ± 1.5 μg/ml (2.3)
Ammonia production	0.20 ± 0.01 μmoles/ml
Germination Index (GI)	116.348 ± 38.02%
Pathogenic potential	
Haemolysis on blood agar	- ve
Protease production	0.17 ± 0.002 U/mg
Biofilm production	- ve at 37 °C, weak producer at 15 °C
Antibiotic susceptibility test	AZM^−^, AMP^−^, CIP^−^, CHL^−^, E^−^, G^−^, K^−^, P^−^, RIF^−^, S^−^, TE^−^, VA^−^

Values in parentheses indicate ^a^Optimum growth condition, ^b^enzymatic index at 10 °C, ^c^ Siderophore producing index; ^d^ phosphate solubilization index; +: Resistant; −: Sensitive; AZM: 15 mcg, Azithromycin; AMP: 10 mcg, Ampicillin; CIP: 5 mcg, Ciprofloxacin; CHL: 30 mcg, Chloramphenicol; E: 15 mcg Erythromycin; G:10 mcg, Gentamycin; K: 30 mcg, Kanamycin; P: 10 Units, Penicillin-G; RIF: 5 mcg, Rifampicin; S: 10 mcg, Streptomycin; TE: 30 mcg, Tetracycline; VA: 30 mcg, Vancomycin

The nutrients released after the decomposition of polysaccharides tend to leave the agricultural systems due to leaching, surface runoff, and eutrophication [16]. As a result, availability for plant uptake is always questionable; however, the inhabitant PGP bacteria improves nutrient uptake and produces phytohormones aiding the efficiency of applied compost [17]. Hence, LJH 19 was further explored to investigate its PGP potential for additional properties to become a suitable bioinoculant candidate forenhancing soil nutrients at high altitude agro-ecosystems. In the qualitative assay for siderophore production, the LJH19 strain showed orange halo zones at varying temperatures and the best results of the siderophore index of 1.5 at 10 °C (Table 1) (Supplementary Figure S3D). Quantitatively, LJH19 exhibited considerable siderophore production of 85.72 ± 1.06% siderophore unit (psu) at 10 °C. In the absorption spectra, we observed a peak at 292 nm supporting the presence of 2,3-dihydroxybenzoic acid (DHB) in the supernatant (Supplementary Figure S4). A previous study reported that in acidic medium DHB, a phenolic compound consisting of a catechol group absorbs below 330 nm showing two absorption bands with maxima at 254 nm and 292 nm, respectively [18]. DHB is an intermediate involved in the synthesis of catecholate type siderophore [19]. This evidence supports the presence of DHB in the supernatant, indicating the production of catecholate type siderophore by strain LJH19. Siderophore production by PGPB is vital for plant defense. Iron chelation by siderophores suppresses fungal pathogens in the rhizosphere [20]. LJH19 also demonstrated the ability to produce 166.11 ± 5.7 μg/ml of IAA after 72 Hrs of incubation with 200 μg/ml concentration of L-Trytophan at 10 °C (Supplementary Figure S3A), signifying that auxin production occurs through the tryptophan-dependent pathway. Production of phytohormone IAA is essential for plant growth to proliferate lateral roots and root hairs [21]. Qualitative estimation of phosphate solubilization by LJH19 showed positive results at varying temperatures, and the best activity of 2.3 solubilization index was displayed at 10 °C (Table 1). Quantitatively, LJH19 solubilized 44.76 ± 1.5 μg/ml of tri-calcium phosphate at 10 °C after the 5th day of incubation in NBRIP broth (Supplementary Figure S3C). The activity of bacteria decreased pH from 7 to 4.5, indicating the elevation of phosphate solubilization levels. Thus the results suggested, the presence of LJH19 in the compost can deliver available phosphorous to the plants. Since plants cannot uptake inorganic phosphate present in a fixed or precipitated form in the soil, bacteria aids in increasing the availability of soluble P for plant acquisition through solubilization [22]. While performing in-vitro assays for ammonia production, strain LJH19 produced a low level of ammonia (0.20 ± 0.01 μmoles/ml) (Supplementary Figure S3B) after 10 days of incubation in peptone water. Ammonia production by bacteria is yet another PGP feature to increase nitrogen availability [23]. However, these values are relatively low in the case of PGP attributes. In the composting case, ammonia gas released by bacteria is primarily responsible for the pungent smell and loss of organic nitrogen from the compost [24]. The results may therefore suggest that it doesn’t directly benefit the plants but may maintain stable organic nitrogen content in the compost by not converting rich nitrogenous sources into ammonia gas.

The seed germination rate was significantly higher in the treated pea *Pisum sativum* var. Arkel seeds (83.33 ± 15.27%) from the control (66.66 ± 15.27%) (Supplementary Figure S5). The relative seed germination, relative root growth, and relative shoot growth were noticeably increased to 135.55 ± 33.55%, 103.70 ± 33.60%, and 112.78 ± 12.14% respectively, subjected to the treatment of pea seeds with bacterial inoculation. The germination index was further recorded as 116.348 ± 38.02% under the bacterial influence. The LJH19 strain capabilities to produce auxin and siderophore may have positively affected pea seeds’ seed germination. In agreement with our findings, other *Glutamicibacter* strains have also shown PGP traits where a strain *G. halophytocola* KLMP 1580 have significantly promoted the growth of *Limonium sinense* under high salinity stress [25]. In another study, *G. halophytocola* KLMP 1580 was also reported to enhance tomato seedlings’ growth [26]. Another *Glutamicibacter* species, *G. creatinolyticus* was reported as an efficient PGPR with IAA production [27]. Similarly, the closest related genus *Arthrobacter* has also been reported to exhibit excellent PGP attributes, having a potential role in recovering burned soils of holm-oak forests [15, 28, 29].

Owing to the source of LJH19 strain isolation from night-soil, it was mandatory to ensure its safety for humans before declaring it as a suitable candidate as a bioinoculant. Hence, the strain LJH19 was tested for its pathogenicity. In general, any pathogenic bacteria rely on various virulence factors to induce pathogenesis, including adhesion proteins, toxins like hemolysins, and proteases [30]. The initial screening of virulence of LJH19 performed on blood agar showed no hemolytic activity compared to the other tested hemolytic strains MTCC 96, MTCC121, MTCC 43, MTCC 2470 (Supplementary Figure S6A). LJH19 was tested positive for protease activity with an enzymatic index of 12.5 (Supplementary Figure S6B), but, quantitatively LJH19 showed very low protease activity (Table 1).

Furthermore, strain LJH19 was not observed to form biofilm on polystyrene at 37 °C (Supplementary Figure S6C). The adherence of bacteria to the host tissue cells is the initial step to induce the pathogenesis [31]. Therefore, biofilm formation is a notable virulence factor of pathogenic potential and is directly related to the strain’s safety. The LJH19 strain also exhibited antibiotic susceptibility to all the 12 antibiotics tested (Supplementary Figure S6D), (Table 1).

Night-soil composting remains dormant during winters as the temperature goes to sub-zero conditions, and microbial degradation plays an insignificant role in odor formation. However, in the summer months, where the temperature ranges from 5 to 25 °C [1], slow microbial metabolism due to low microbial load produces a strong odor during composting. During this period, night-soil composting can be improved by supplementing it with a psychrotrophic bacterial consortium. Owing to the survivability at 4 °C and efficient hydrolytic activity at varying temperatures (best activity at 10 °C), non-pathogenicity, and PGP potential, strain LJH 19 qualifies a potential bio-inoculant candidate for the preparation of a psychrotrophic consortium for accelerated degradation and quality improvement of NSC. Further, the whole genome sequencing, data mining, and comparative genomics of strain LJH19 bacterium were explored to obtain genetic bases on its potential to be a safe bio-inoculant for the consortia and to investigate the niche-specific gene repertoire.

### Genomic features of strain LJH19

RS hierarchical genome assembly was performed as described previously in Kumar et al. [32]. The assembly generated a draft genome (4 contigs) of 3,602,821 bp (N50 read length 2,610,692) with 59.60% GC content with average mean coverage of 153 X (Supplementary Table S1) (GenBank accession number: SPDS00000000). The NCBI Prokaryotic Genome Annotation Pipeline (URL: www.ncbi.nlm.nih.gov/genome/annotation_prok) prediction revealed a total of 3517 genes out of which 3396 were protein-coding genes (covering 96.56% of the genome) and 99 RNA genes (30 rRNAs, 66 tRNAs, and 03 other RNA genes). There was no plasmid DNA in the genome of LJH19, as evident by no observation of bands in agarose gel electrophoresis after the plasmid isolation. Additionally, in silico analysis with PLSDB web-based tool supported no plasmid existence in the genome of LJH19.

### Whole genome-based phylogenetic assessment and genome relatedness

**Fig. 2:**
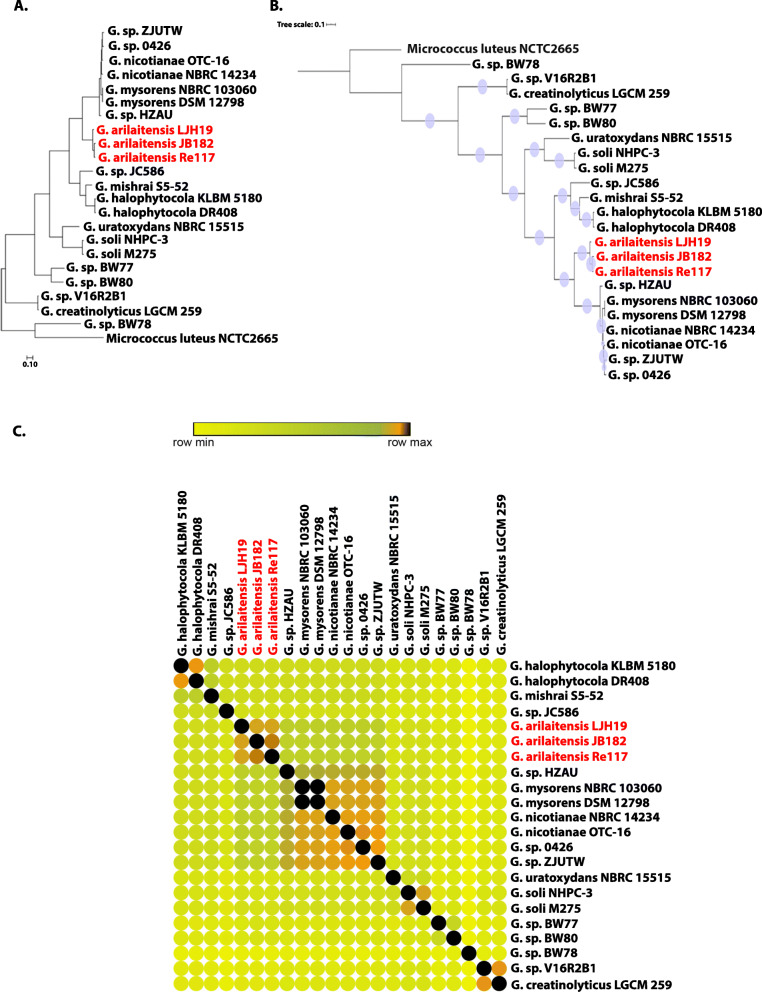
Phylo-taxono-genomics of *Glutamicibacter arilaitensis* LJH19. **a** 16S rRNA gene phylogeny obtained from all available *Glutamicibacter* strains. **b** ML-based phylogenomic tree construction obtained from the whole proteome information of the strains of genus *Glutamicibacter*. Violet color circle at each node represents corresponding bootstrap values. **c** OrthoANI similarity matrix created with morpheous, red color represents the maximum values, yellow color represents the minimum values, green color is the intermediate values, and orange color represents the cutoff value for species demarcation (95% similarity)

Figure The 16S rRNA gene phylogenetic tree clustered the strains *G*. *arilaitensis* LJH19, *G*. *arilaitensis* JB182, and type strain *G*. *arilaitensis* Re117^T^ into a single clade (Fig. 2a). The phylogenetic analysis using PhyloPhlAn pipeline with 400 conserved gene sequences of *Glutamicibacter* sp. was also congruent in clustering the strains *G. arilaitensis* LJH19, JB182, and type strain Re117^T^ into a single clade (Fig. 2b). The average nucleotide identity (ANI) between genomes was calculated using orthoANI to differentiate species at a 95% similarity threshold. The ANI matrix also suggests the genome similarity of the strain LJH19 to *G*. *arilaitensis* Re117^T^ and JB182 (Fig. 2c).

### Pan-genome analysis and chromosomal map

**Fig. 3:**
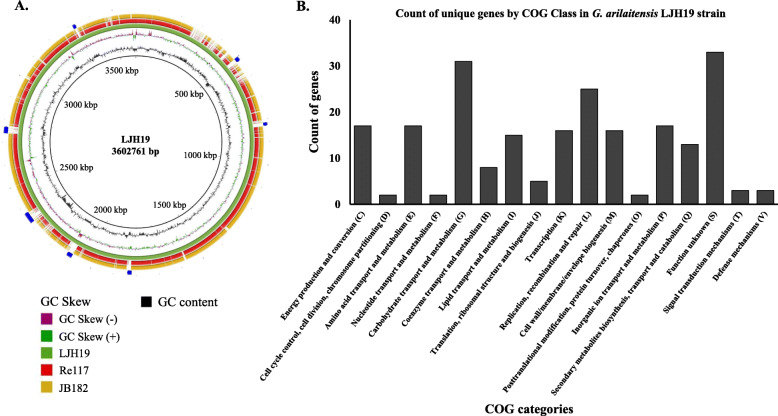
Circular genome representation and clusters of orthologous groups (COG) classes of *G. arilaitensis* LJH19. **a** BRIG implementation across the three closely related strains, including type strain *G. arilaitensis* Re117^T^, *G. arilaitensis* JB182, and *G. arilaitensis* LJH19, which resulted in identifying the unique genomic region across the isolate LJH19. **b** COG classes were identified for the unique genes retrieved from pan genome analysis for *G. arilaitensis* LJH19 genome

**Fig. 4:**
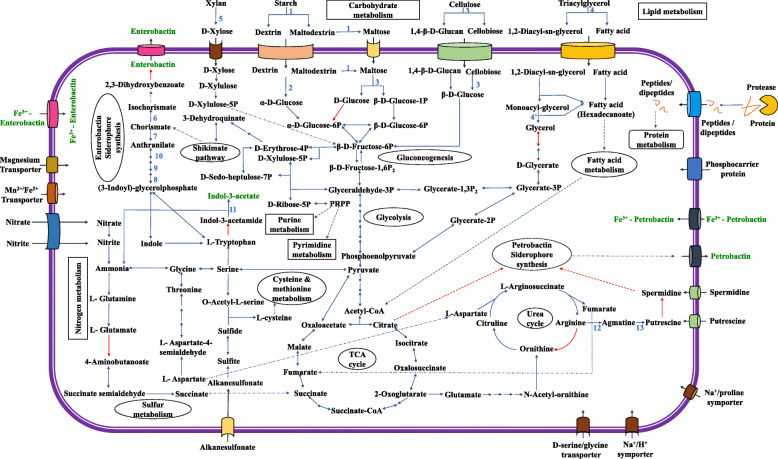
Schematic representation of the predicted genes encoding catabolic activities, transport and plant-growth promotion in *G. arilaitensis* LJH19. The selected key genes involved in the pathway indicated by blue arrows are: 1, amylase; 2, Oligo-1,6-glucosidase; 3, Beta-glucosidase; 4, Triacylglyceride lipase; 5, monoacylglycerol lipase; 6, Anthranilate synthase component I (TrpE); 7, Anthranilate phosphoribosyl transferase (TrpD); 7,Phosphoribosyl anthranilate isomerase (TrpF); 9, Indole-3-glycerol phosphate synthase (TrpC); 11, Isochorismate synthase (menF); 12, Isochorismatase; 13, Amidase; 14, Argininosuccinate lyase (argH); 15, Arginine decarboxylase (speC); 16, Agmatinase (speB); 17, Polyamine aminopropyl transferase (speE); 18, Ornithine decarboxylase (speC). Core metabolic enzymes indicated in the pathway by blue arrows are listed in supplementary Table S2. Red arrows indicate enzymes missing in the metabolic pathway. Multistep pathways are denoted with dotted lines

Roary run for the strains forming a clade with the type strains of *G. arilaitensis* and LJH19 resulted in a pan-genome of 9892 genes. A total of 634 genes were found to be core genes, whereas the gene clusters specific to the strain LJH19, Re117^T^, and BJ182 were 1740. A total of 217 genes were specific to the strain LJH19. Chromosomal map showing the unique genomic regions across the strain LJH19 depicts the uniqueness of the strain LJH19 (Fig. 3a). All the strain-specific gene from LJH19 classified by eggNOG falls in several clusters of orthologous groups (COG) categories (Fig. 3b). A list of the unique gene, function, and COG classification is reported in Supplementary Table S2. Based on the annotation and unique genes data, an image illustrating a schematic representation of predicted genes associated with catabolic activities, transport, and plant growth promotion of the genome of LJH19 was generated (Fig. 4).

### Genomic insights into the safety of LJH19

Virulence is a characteristic of pathogenicity which confers the ability to initiate and sustain infection for the organism. The occurrence of such determinants at the genetic level makes the organism potentially pathogenic with the ability to circulate such genes in the bacterial population [33]. The LJH19 genome was analyzed for the presence of virulence factors using the virulence factors of the pathogenic bacteria database (VFDB) [34]. We found LJH19 contained few genes designated as virulence factors (Supplementary Table S3). Putative chain-fatty acid-CoA ligase (FadD13) is required for maintenance of the appropriate mycolic acid composition and envelope permeability in *Mycobacterium* sp. and is involved in the fatty acid biosynthesis pathway, a portion of lipid metabolism [35]. We found 12 hits for FadD13 gene in LJH19 genome. To further confirm these results, the LJH19 genome was assessed for its pathogenic potential by PathogenFinder [36]. The web-based tool identifies the genome and provides a probability measure for the test strain to be pathogenic for humans. The predicted results identified LJH19 as a non-human pathogen with an average probability of 0.356 (Supplementary Table S4). None of the putative virulence or pathogenic genes were identified in the tested genome. The LJH19 genome was further screened for the antibiotic resistance genes using CARD pipeline [37]. Evaluation of resistomes in the genome revealed 208 hits to drug classes (Supplementary Table S5). The AMR gene family identified were beta-lactamase, glycopeptide resistance gene cluster, trimethoprim resistant dihydrofolate reductase, tetracycline-resistant ribosomal protection protein, sulfonamide resistant, rifampin phosphotransferase, resistance-nodulation-cell division (RND) antibiotic efflux pump major facilitator superfamily (MFS), antibiotic efflux pump, resistance-nodulation-cell division (RND) antibiotic efflux pump, etc. (Supplementary Table S5). These results indicated resistance against drugs such as tetracycline, rifamycin, quinolones, macrolactams, macrolides, aminoglycoside, lincosamide, carbapenem, cephalosporin, pyrazinamide, etc. (Supplementary Table S5). However, LJH19 displayed a negative resistance phenotype to all the 12 antibiotics tested in the in vitro assays (Table 1; Figure S6D). These results suggested the safety of strain LJH19.

### Genomic insights into the cold adaptation of LJH19

Psychrotrophic bacteria isolated from high altitude ecosystems have unique adaptations to survive in a cold environment maintaining their growth and metabolism [32]. LJH19 was isolated from a night-soil compost of the high-altitude ecosystem of Lahaul valley in northwestern Himalaya that experiences extreme temperature variations [1]. Psychrotrophic bacteria sustain these extreme factors with unique cold-adapted proteins active at low temperatures. There are reports on such cold-associated genes in the genome of cold-adapted bacteria [9, 32, 38]. LJH19 genome also predicted several cold-associated genes encoding for proteins responsible for cold-active chaperons, general stress, osmotic stress, oxidative stress, membrane/cell wall alteration, carbon storage/ starvation, DNA repair, Toxin/Antitoxin modules were identified across the genome (Table 2). This genomic evidence supports the versatility of the LJH19 strain to survive in a broad temperature range of 4 to 37 °C. Bacteria inhabiting high altitude regions are also prone to the accumulation of reactive oxygen species (ROS) such as hydrogen peroxide, superoxide’s, and hydroxyl radicals, and to prevent the damage caused by these radical bacteria synthesize antioxidative enzymes [9, 32, 38, 39]. Similarly, the LJH19 genome predicted multiple genes encoding antioxidant enzymes such as catalase, superoxide dismutase, thioredoxin, and Thioredoxin-disulphide reductase (Table 2). Additionally, the genome of the strain LJH19 also predicted genes encoding proteins involved in DNA repairs such as Recombinase, DNA repair protein RadA, DNA integrity scanning protein DisA, and DNA repair protein RecN (Table 2) that may aid in the robust feature of strain LJH19 in surviving the extreme conditions.

**Table 2:** Genes encoding known cold & stress response and DNA repair proteins as predicted in the genome of *G. arilaitensis* LJH19

Category and GenBank ID	Description	Category and GenBank ID	Description
**Cold active chaperones**		**Osmotic Stress/ Oxidative stress**	
TFH54768, TFH56297, TFH57075	Cold-shock protein	TFH56153	Glycine betaine ABC transporter substrate-binding protein
TFH56633	Co-chaperone GroES	TFH56253	Sarcosine oxidase subunit beta family protein
TFH56634, TFH54762	Chaperonin GroEL	TFH56254	Sarcosine oxidase subunit delta family protein
TFH57159	Molecular chaperone DnaJ	TFH56255	Sarcosine oxidase subunit alpha family protein
TFH54768, TFH56297, TFH57075	Cold-shock protein	TFH56256	Sarcosine oxidase subunit gamma family protein
TFH56633	Co-chaperone GroES	TFH56949	Superoxide dismutase
TFH56416	Molecular chaperone DnaK	TFH57402, TFH54880	Catalase
TFH56424	ATP-dependent chaperone ClpB	TFH56696, TFH56988, TFH56162	OsmC family peroxiredoxin
TFH57532	Heat shock protein HslJ **/** META domain-containing protein	TFH55537	Organic hydroperoxide resistance protein
TFH56416	Molecular chaperone DnaK	TFH55668, TFH56321, TFH56523, TFH54754, TFH55543	Thioredoxin
TFH56424	ATP-dependent chaperone ClpB	TFH55669	Thioredoxin-disulfide reductase
**Carbon storage/starvation**		TFH55955, TFH57498	Thioredoxin-dependent thiol peroxidase
TFH54400	Carbon starvation protein A	TFH56887	Thioredoxin family protein
TFH56944, TFH57627, TFH57801	1-acyl-sn-glycerol-3-phosphate acyltransferase	TFH54908	Thioredoxin domain-containing protein
**Membrane/cell wall alteration**		TFH56279	Sodium/proline symporter PutP
TFH55154	3-oxoacyl-ACP synthase III	TFH54956	Na^+^/H^+^ antiporter NhaA
TFH57491	Phytoene desaturase	TFH54753	Trehalose-6-phosphate synthase
TFH57492	Phytoene/squalene synthase	TFH54752	Trehalose-phosphatase
**General Stress response**		**DNA repair**	
TFH55892, TFH56176, TFH56200, TFH56262, TFH57611, TFH55142, TFH54651, TFH55629	Universal stress protein	TFH54856, TFH54452	Recombinase family protein
TFH57700, TFH57339	GlsB/YeaQ/YmgE family stress response membrane protein	TFH55191	Recombinase RecA
TFH54980, TFH56483	Serine/threonine protein kinase	TFH57358	Tyrosine recombinase XerC
TFH57322	Peroxide stress protein YaaA	TFH57661	Site-specific tyrosine recombinase XerD
TFH54953	50S ribosomal protein L25	TFH57734	Recombinase
TFH57081	SOS response-associated peptidase	TFH54405	ATP-dependent DNA helicase RecQ
**Toxin/Antitoxin modules**		TFH55567	DNA repair protein RadA
TFH54837	Type II toxin-antitoxin system prevent-host-death family antitoxin	TFH57167	DNA repair protein RecO
TFH54532	Type II toxin-antitoxin system VapB family antitoxin	TFH55568	DNA integrity scanning protein DisA
TFH54610	Type II toxin-antitoxin system HipA family toxin	TFH57664	DNA repair protein RecN
TFH56658	Type II toxin-antitoxin system Phd/YefM family antitoxin	TFH57433	ATP-dependent DNA helicase RecG
TFH57154	Type II toxin-antitoxin system PemK/MazF family toxin	TFH56747	ATP-dependent DNA helicase UvrD2
TFH57340	Toxin component of a toxin/antitoxin system	TFH57103	Holliday junction branch migration protein RuvA
TFH55640	Serine/threonine-protein kinase	TFH57104	Holliday junction branch migration DNA helicase RuvB
		TFH57121	Holliday junction resolvase RuvX

Genomic insights on nutritional versatility and adaptation to environmental stresses have been documented previously for other *Glutamicibacter* strains [12]. Similar to genomic evidence on cold adaptation of LJH19, adaptation towards salt tolerance, oxidative and osmotic stress tolerance from varied ecological habitats such as cheese, coastal halophyte, rhizospheric soil, and coral *Favia veroni* have been reported previously (Supplementary Table S6) [26, 40–42]. Likewise, multiple reports on genomic evidence to support physiological adaptation for varied stress adaptations in the nearest genus *Arthrobacter* are also available [14, 15, 29, 43] (Supplementary Table S6). The current study and other genomic insights supported the niche-specific adaptational strategies of the genus *Glutamicibacter* in the varied ecological habitats.

### Genomic insights into the hydrolytic potential of LJH19

The biodegradation of complex polysaccharide molecules by bacteria requires a cocktail of enzymes to depolymerize it to oligosaccharides and monomer sugars [44]. The genome of LJH19 showed the occurrence of multi copies of genes encoding for proteins responsible for the metabolism of a wide variety of complex polysaccharides like cellulose, starch, and xylan. Similar to the finding in LJH19, the genome of *G. arilaitensis* Re117^T^ strain also has been reported encoding genes involved in protein and lipid degradation [41] (Supplementary Table S6). The key enzymes encoded in the LJH19 genome are beta-glucosidase, alpha-amylase, beta-xylosidase, pullulanase, oligo-1,6-glucosidase, and glycosidases associated with the degradation of polysaccharides (Fig. 4; Table 3; Supplementary Table S5). These findings endorse the experimental evidence of LJH19 showing enzymatic activities against complex polysaccharides that aids in the improved composting process. For the utilization of cellulosic substrates, psychrotrophic bacteria requires the ABC transporters specific for the hydrolytic product, such as cellobiose, cellodextrin, β-D-Glucose. Cellulases such as beta-glucosidase cleave the β-(1,4)-glycosidic linkages within the cellulose polymer releasing cellobiose, glucose, and cellodextrin, which are then transported inside the cell via specific transporters [45]. LJH19 genome also predicted genes encoding proteins that are components of transporter complexes engaged in the recognition and transport of monosaccharides and oligosaccharides such as maltose/maltodextrin, maltooligosaccharide, and cellobiose and transporters for hydrolyzed proteins (Table 3; Supplementary Table S6).

**Table 3:** Genes encoding proteins involved in catabolic activity, plant growth promoting activity, transport and cold adaptation predicted in the genome of *G. arilaitensis* LJH19

Category and GenBank ID	Description	Category and GenBank ID	Description
**Catabolic activity**		**Plant Growth Promoting activity**	
TFH56992, TFH56993	ATP-dependent Clp protease proteolytic subunit	TFH55608, TFH57060	amidase
TFH56994	ATP-dependent Clp protease ATP-binding subunit ClpX	TFH56909	Anthranilate synthase component I
TFH54429	Putative esterase	TFH56082	Nitrite reductase [NAD(P)H]
TFH56413	pullulanase-type alpha-1,6-glucosidase	TFH56083	nitrite reductase (NAD(P)H) small subunit
TFH57366	trypsin-like serine protease	TFH56086	nitrite reductase
TFH56438	MarP family serine protease	TFH57000	nitrite/sulfite reductase
TFH57809	Alpha-amylase	TFH55619	nitrate reductase
TFH54465	alpha/beta fold hydrolase	TFH56511	Isochorismate synthase
TFH54414	Beta-glucosidase	TFH57125	chorismate synthase
TFH54614	Xylose isomerase	TFH57182	Anthranilate phosphoribosyl transferase
**Transporters**		TFH57559, TFH55965	Isochorismatase family protein YecD
TFH56366	spermidine/putrescine ABC transporter substrate-binding protein	TFH55761	Acetylornithine aminotransferase
TFH54902, TFH55612, TFH56015, TFH56106, TFH56479, TFH56632, TFH56864, TFH57051, TFH57718	amino acid permease	TFH54544	alkaline phosphatase
TFH56389	amino acid ABC transporter ATP-binding protein	TFH54792	Inositol-1-monophosphatase
TFH55562	phosphate ABC transporter ATP-binding protein	TFH56913	tryptophan synthase subunit beta
TFH55563	phosphate ABC transporter permease PstA	TFH56914	tryptophan synthase subunit alpha
TFH55564	phosphate ABC transporter permease subunit PstC	TFH56912	indole-3-glycerol phosphate synthase TrpC
TFH55565, TFH57701	phosphate ABC transporter substrate-binding protein PstS	TFH55232	Ornithine carbamoyltransferase
TFH55134	phosphate/phosphite/phosphonate ABC transporter substrate-binding protein	TFH56276	ornithine decarboxylase
TFH55132	phosphonate ABC transporter, permease protein PhnE	TFH55236	Argininosuccinate lyase
TFH55133	phosphonate ABC transporter ATP-binding protein	TFH55205	Phosphoribosyl anthranilate isomerase PriA
TFH56773	peptide ABC transporter substrate-binding protein	TFH56212	Formimidoyl glutamase* (Arginase)
TFH54587	aliphatic sulfonate ABC transporter substrate-binding protein	TFH54789	agmatinase
TFH54588	ABC transporter permease	TFH55596	inorganic diphosphatase
TFH55268	short-chain fatty acid transporter	TFH56916	glutamate synthase subunit beta
TFH56023	D-serine/D-alanine/glycine transporter	TFH57767	glutamate synthase large subunit
TFH55620, TFH56087	MFS transporter (nitrate)	TFH56292	FMN-binding glutamate synthase family protein
TFH56177	gluconate permease	TFH56820	Glutamine synthetase
TFH55044	iron-enterobactin ABC transporter permease		
TFH55045	Fe^(3+)^-siderophore ABC transporter permease		
TFH54561, TFH54802	siderophore-interacting protein		
TFH54562	Fe^2+^-enterobactin ABC transporter substrate-binding protein		

Furthermore, genes such as triacylglycerol lipase were also predicted associated with fatty acid degradation (Fig. 4; Supplementary Table S6). Within the cells, enzymes (like beta-glucosidase, oligo-1,6-glucosidase, alpha-amylase) attack the polysaccharides producing smaller oligosaccharides and monomeric sugars. Finally, the monomeric sugars like glucose go into the glycolysis pathway and ultimately to the TCA cycle generating energy for cellular growth [46]. For better understanding, an overview of a similar mechanism has been represented in the LJH19 cell based on the prediction of genes encoding critical proteins for polysaccharides metabolism and transporters from the genome (Fig. 4).

### Genomic insights into plant growth-promoting potential of LJH 19

A series of genes encoding enzymes related to PGP traits predicted in the LJH19 genome were amidase, isochorismate synthase, isochorismatase family protein YecD, nitrite reductase, nitrate reductase, and alkaline phosphatase (Table 3). Quantitatively LJH19 strain showed auxin production by utilizing L-tryptophan, and it got endorsed by the genomic evidence that predicted tryptophan dependent pathway utilizing L-tryptophan (Fig. 4; Table 3). Genes encoding amidase, N-acetyltransferase, and acetaldehyde dehydrogenase for auxin synthesis were predicted in the LJH19 genome (Table 3; Fig. 4). Auxin plays a vital role in the development of lateral plant roots and stem elongation [21]. The experimental studies also have shown remarkable siderophore production by LJH19 strain that is an important plant defense, suppressing fungal pathogens in the rhizosphere [20]. Upon genome mining, genes involved in the synthesis of polyamines (PAs), putrescine (Put), and spermidine (Spd) were also identified in the LJH19 genome (Table 3; Supplementary Table S6). In bacteria, these active molecules are involved in siderophore biosynthesis, improve the survival rate in freezing conditions, and stabilize spheroplasts and protoplasts from osmotic shock [47].

As discussed earlier, experimental evidence suggested that LJH19 is involved in the catecholate type siderophore production. The genomic insights further strengthened these findings by predicting the genes involved in enterobactin and petrobactin’s biosynthesis (Table 3). These results indicate that LJH19 has the potential to produce a wide variety of siderophores. Most of the enzymes involved in enterobactin biosynthesis were predicted except the genes involved in converting 2,3-Dihydro-2,3-dihydroxybenzoate to enterobactin, marked as a red arrow in Fig. 4 (Supplementary Table S2). LJH19 genome also predicted the genes encoding the transporters required for the import and export of synthesized enterobactin. In respect to petrobactin’s biosynthesis, spermidine molecules are used for synthesis using citrate backbone [48].

Further, genes encoding the transporters required to import and export both synthesized enterobactin and petrobactin and transporters for hydroxamate type siderophores were predicted in the genome (Supplementary Tables S2, S6). In addition to auxin and siderophore, the LJH19 genome also predicted few genes encoding phosphatases, inositol-phosphatases, and gluconate permease (Fig. 4; Table 2; Supplementary Table S6) involved in phosphate metabolism. LJH19 strain has also been noted to carry genes involved in nitrate/nitrite transport pathways, including the genes associated with denitrification and nitrate reduction like nitrite reductase and nitrate reductase (Fig. 4; Table 3; Supplementary Table S6). Nitrite reductase encoded by the NirD gene converts nitrite to ammonium and further converted to glutamate by glutamate synthetase for amino acid metabolism (Fig. 4). Thus, LJH19 may deliver plants with available nitrogen sources via enzymatic conversion.

The cold-tolerant LJH19 has shown potential PGP properties physiologically, and genomic evidence has supported the function. Similar genomic insights for saline tolerant strain *G. halophytocola* KLBMP 5180 has also been reported to carry the genes related to PGP, such as siderophores and spermidine biosynthesis [26]. Like LJH19, KLBMP 5180 also harbored genes such as agmatinase, spermidine synthase, siderophore ABC transport system ATP-binding protein, siderophore ABC transporter substrate-binding protein (Supplementary Table S6). Similarly, *G. halophytocola* DR408 genome also carried PGP genes involved in siderophore production and phosphate solubilization [42] (Supplementary Table S6). Although few reports of genomic evidence of PGP potential of *Glutamicibacter* species are available in the literature, the closest related genus *Arthrobacter* has multiple reports on genetic evidence of PGP traits [15, 29, 49, 50]. Among the *Arthrobacter* species, *A. agilis* L77 [15] and *A. alpinus* R3.8 [29] possessing PGP traits such as phosphate solubilization, IAA, and ammonia production are also reported for cold adaptation.

### Genomic insights into secondary metabolic gene cluster of LJH19

**Fig. 5:**
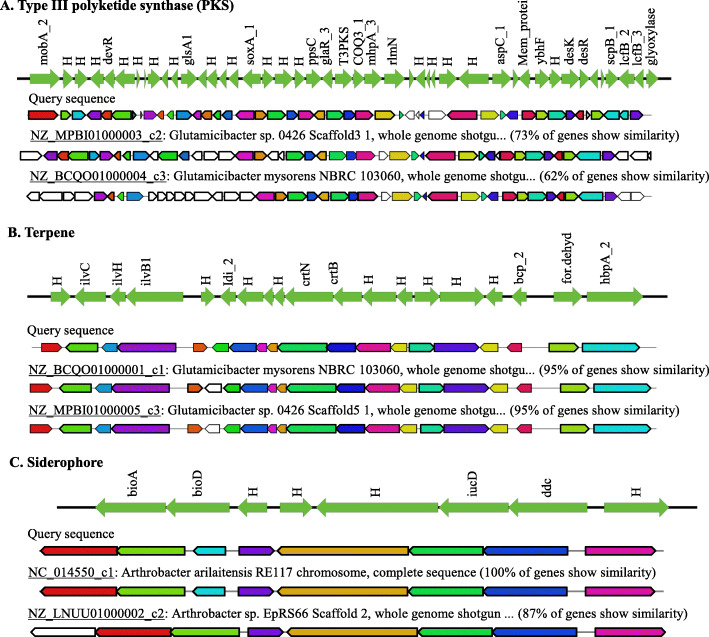
Identification of biosynthetic gene clusters by antiSMASH v5.0. in *G. arilaitensis* LJH19 genome. The predicted gene cluster showed a significant hit with other strains of genus *Glutamicibacter*. H represents the hypothetical gene cluster annotated by prokka v1.14.6. The arrow’s direction represents the forward (5′ → 3′) and reverse orientation of the gene cluster

Phylum actinobacteria are very well known for producing a variety of secondary metabolites [51]. Secondary metabolites gene clusters search using antiSMASH v5.0 resulted in identifying three biosynthetic gene clusters, namely type III polyketide synthase (PKS), terpene, and siderophore (Fig. 5). Type III PKS are involved in synthesizing numerous metabolites and have various biological and physiological roles, such as antimicrobials and defense systems in bacteria [52]. Such a gene cluster with a probable biological function in producing antimicrobial metabolites favors LJH19 as a PGP bacterium for being a biological control agent against phytopathogens [53]. The presence of a carotenoid gene cluster supports the indicative yellow color of the LJH19 colonies. Besides pigmentation, carotenoid’s major function in bacteria is to protect the cell from UV radiations, oxidative damage and modify membrane fluidity [54]. Siderophore production is another attribute that has several ecological applications in plant growth promotion and acts in plant defense against various pathogens [55]. Prediction of the siderophore gene cluster in the genome of LJH19 endorses the experimental evidence of catecholate type siderophore production by LJH19. It supports the presence of several siderophores associated genes in the genome of LJH19.

## Conclusion

Night-soil compost is a rich nutrient source and, when supplemented to the soil, increases its fertility. In this study, *G. arilaitensis* LJH19 isolated from NSC demonstrated the ability to hydrolyze complex polysaccharides richly found in night-soil and agricultural residues like starch, cellulose, and xylan. The bacterium survived extreme cold conditions and exhibited several PGP traits such as auxin production, siderophore production, and phosphate solubilization at low ambient temperature. The strain displayed its capabilities as a safe bacterium by demonstrating negative hemolysis and biofilm formation. Genomic search reinforced the bacterium’s safety with the absence of any virulence and antibiotic resistance genes. A comprehensive genomic analysis predicted and excavated key genes related to cold adaptation, polysaccharide metabolism, and plant growth promotion. These results indicated *G. arilaitensis* LJH19 may serve as a safe bioinoculant and may contribute to efficient psychrotrophic bacterial formulations for improved night-soil degradation and soil enrichment PGP attributes. To the best of our knowledge, the current study is the pioneering scientific intervention addressing NSC’s issue in the high Himalayas.

## Materials and method

### Sampling source, strain isolation, and hydrolytic potential

NSC samples were collected from the collection chamber of the night-soil composting toilet, locally termed as ‘*ghop*’ of Jundah village (32.64°N 76.84°E) of Lahaul valley (Fig. 1a). The samples were collected from the core of the compost pile in sterile plastic bottles using stainless steel spatula in triplicates and stored in an icebox containing ice packs. The samples were then immediately transported to the laboratory and processed. The temperature was noted at the time of sampling by inserting the handheld digital thermometer (MEXTECH, India) into the compost pile’s core. Air-dried solid sample was mixed with Milli-Q water at a ratio of 1:10 vortexed and kept overnight to check pH and electrical conductivity (EC) using digital pH and EC meter (Eutech, India). The samples were dried at room temperature, finely grounded, and sieved to analyze total available nitrogen, phosphorus, and potassium. All the chemical analysis was performed as per the standard methods for testing compost materials [56].

The bacterial strain LJH19 was isolated from NSC samples while screening for potential psychrotrophic hydrolytic bacteria. The isolation was carried out using serial dilution and spread plate methods on nutrient agar (NA) medium (HiMedia) at 10 °C. The bacterial isolation was performed in Class II, Type A2 Biological Safety Cabinet (Thermo Scientific, US). The optimum growth conditions of the LJH19 strain were determined by incubating the culture at various temperatures (4–50 °C), NaCl concentration (1–10%), and pH (2 to 10) range. The production of hydrolytic enzymes by the LJH19 strain was initially screened using a plate assay method. An exponentially grown culture of LJH19 were spot inoculated on Carboxymethylcellulose (CMC) agar [57], Starch agar (Hi-Media), Xylan agar [58], and Tributyrin agar (Hi-Media) plates and incubated at 10 °C for 48 h. The clear halo zones around the colony indicated positive results. The enzymatic index (EI=Diameter of the halo of hydrolysis/Diameter of the colony) was calculated as described previously [59]. For quantitative estimation of polysaccharide degrading enzymes viz. cellulase, xylanase, and amylase, the microplate-based 3, 5-dinitrosalicylic acid colorimetry method was followed using 1% (w/v) carboxymethylcellulose (CMC), 1% birchwood xylan (HiMedia), and 1% soluble starch (HiMedia) as the substrate [60].

### Haemolysin and protease assay, biofilm formation and antibiotic susceptibility profile

For assessment of pathogenic potential, we assayed LJH19 for protease and hemolysin activity using a plate assay method [61]. The strains *Staphylococcus aureus subsp. aureus* (MTCC 96), *Bacillus subtilis* (MTCC121), *Escherichia coli* (MTCC 43), *Micrococcus luteus* (MTCC 2470) were used as a positive control for hemolytic activity. Hemolytic activity was interpreted according to Buxton [62].

Biofilm formation was evaluated according to Basson and Igbinosa [33, 63] with slight modifications. The adhered cells were stained with 200 μL of 0.5% crystal violet for 10 min. The optical density (OD) readings from respective wells were obtained at 595 nm. The cutoff OD (ODc) for the test was set using the formula (Mean OD of negative control +3x Standard deviation), and results were interpreted as previously described [63]. The wells containing only TSB broth (200 μL) served as negative control while the wells containing *Staphylococcus aureus subsp. aureus* (MTCC 96), *Bacillus subtilis* (MTCC121), *Escherichia coli* (MTCC 43), *Micrococcus luteus* (MTCC 2470) were used as a positive control. The test organisms were characterized as non-biofilm producers (OD < ODC), weak (ODC < OD < 2ODc), intermediate (2ODc < OD < 4ODc), and strong (OD > 4ODc).

Antibiotic susceptibility profiling was carried out by using the Kirby-Bauer method [64]. The antibiotic discs (HiMedia) used were 15 mcg, Azithromycin (AZM); 10 mcg, Ampicillin (AMP); 5 mcg, Ciprofloxacin (CIP); 30 mcg, Chloramphenicol (CHL); 15 mcg Erythromycin (E); 10 mcg, Gentamycin (G); 30 mcg, Kanamycin (K); 10 Units, Penicillin-G (P); 5 mcg, Rifampicin (RIF); 10 mcg, Streptomycin (S); 30 mcg, Tetracycline (TE); 30 mcg, Vancomycin (VA). The plates were incubated at 10 °C for 48 h. Zones of clearance were measured in millimetres (mm) and interpreted as Resistant, Intermediate, or Sensitive using the manufacturer’s guidelines (HiMedia).

### Plant growth-promoting (PGP) attributes

Indole acetic acid (IAA) production by LJH19 was studied according to Goswami et al. [65] by supplementing the nutrient broth (100 ml) with L-Trytophan (200 μg ml − 1). The IAA test medium inoculated with strain LJH19 was incubated at 10 °C, and the colorimetric assay for detection of IAA was performed at room temperature. Siderophore production and phosphate solubilization by LJH19 has initially screened on Chromeazurol S (CAS) agar [66] and Pikovskya’s agar (HI Media) at 10 °C. Siderophore producing index (SI) and phosphate solubilization index (PSI) was calculated by dividing zone size by colony diameter.

Quantitative estimation of siderophore was done using CAS-shuttle assay [65] by growing LJH19 in iron-free CAS-broth (pH 6.8) at 10 °C at 150 rpm. To determine siderophore’s chemical nature, we examined the absorption maxima (ʎmax) of cell-free supernatant in UV-3092 UV/Visible spectrophotometer. Ammonia production was quantified spectrophotometrically [67]. LJH19 was grown in peptone water at 10 °C for 10 days at 150 rpm. Inorganic phosphate solubilization was estimated by the vanado-molybdate method [68] using NBRIP broth containing 0.5% tricalcium phosphate (TCP).

Seed germination activity of strain LJH19 was carried out using pea seeds (*Pisum sativum* var. Arkel). The bacterium was grown in nutrient broth for 48 h at 10 °C and centrifuged at 10000 rpm to obtain a cell pellet. The pellet was then resuspended in sterile distilled water. Pea seeds were surface sterilized using 5% sodium hypochlorite for 10 mins, followed by several washes with sterilized distilled water. The surface-sterilized seeds were treated with bacterial suspension for 10 mins and allowed to dry under aseptic conditions. Five seeds per pot were then sown in moist sterile vermiculite. The seeds treated with sterile distilled water with no bacterial inoculant served as the control. The pots were then incubated in a controlled growth chamber with three replicates under mixed incandescent and fluorescent illumination of 550 μmol photon/m^2^/s with a 16/8-h light/dark cycle at 25 ± 2 °C and 40 to 50% relative humidity for 7 days. The number of bacterial cells per seed was approx. 10^8^ CFU determined using serial dilution method [69]. The germination index was then calculated using the equation as described by Mondal et al. [70].

### Strain identification, genome sequencing, annotation, phylo-taxono-genomics, and gene content analysis

The genomic DNA was extracted using the conventional CTAB method [71] and for identification, partial 16S rRNA gene sequencing was performed using 27F and 1492R primers as described previously [72]. Plasmid DNA isolation was performed using PureLink® Quick Plasmid Miniprep Kit (Invitrogen, US).

To provide a genetic basis to the experimental evidence, we performed whole-genome sequencing using PacBio RS-II (Pacific Biosciences, US) as previously [39, 43]. The draft genome sequence was deposited in NCBI GenBank with accession number SPDS00000000. Strain identification was done at the species level using EzTaxon (https://www.ezbiocloud.net/identify). Manual curation of the genomes from a public repository for the closest match was performed with NCBI Genome (https://www.ncbi.nlm.nih.gov/genome/?term=Glutamicibacter). Genome quality was assessed using CheckM v1.1.2 [73] in terms of its completeness and contamination present. For the identification of plasmid DNA, in silico analysis was performed using Mash (v 2.1.1) distance search against the PLSDB database with the PLSDB web-based tool (v 0.4.1–6) [74].

Strain phylogeny was assessed using the 16S rRNA gene sequence and whole-genome phylogeny using PhyloPhlAn v0.99 [75]. For the 16S rRNA gene phylogenetic tree construction, all the full-length 16S rRNA reported strains of genus *Glutamicibacter* were used. *Micrococcus luteus* (Nucleotide accession No: AF542073) was used as an outgroup. The 16S rRNA gene phylogenetic tree was constructed using ClustalX v2.1 [76] and FastTree v2.1.8 [77] with default parameters. Whole-genome phylogeny was constructed using PhyloPhlAN (It uses a 400 most conserved gene across bacterial domains and constructs its phylogeny) (Supplementary Table S1). The assembled scaffolds’ functional annotation was performed using the Best-placed reference protein set, GeneMarkS+ version 4.3, with NCBI’s Prokaryotic Genome Annotation Pipeline (PGAP) [78]. Additional annotation and the manual review were performed using prokka v1.14.6 [79] and JGI Prokaryotic Automatic Annotation Pipeline [80]. Data mining across the genome of LJH19 was carried out to identify potential genes for endorsement of its potential safe psychrotrophic bio-inoculant candidate as described earlier [32, 38]. The orthoANI v1.2 [81] was performed to infer the taxonomic relatedness of the strain LJH19. ANI value matrix obtained was used for generating heatmap using the webserver of Morpheus (https://software.broadinstitute.org/morpheus). Further, 10 strains forming a clade were considered for pan-genome analysis with a 95% cutoff using Roary v3.6.0 [82]. The unique gene present in the strain LJH19 was fetched and annotated with eggNOG mapper v1 (http://eggnogdb.embl.de/app/home#/app/home) [83]. Chromosomal maps for comparing the closely related strains and visualization of the unique genomic region across the strain LJH19 to the type strain RE117^T^ and JB182 are marked in the figure (Fig. 3) [84].

To identify the biosynthetic gene clusters (BGCs) in the genome of LJH19, we used a web-based server of antiSMASH v5.0 (https://antismash.secondarymetabolites.org/#!/start) [85] and a cluster image of the identified biosynthetic gene was prepared with EASYfig v2.2.2 [86]. The putative virulence factors were identified by BLAST against virulence factor database (VFDB) with default parameters (http://www.mgc.ac.cn/VFs/main.htm) [34]. The pathogenic potential of LJH19 was also assessed using the PathogenFinder 1.1 web service under automated mode (https://cge.cbs.dtu.dk/services/PathogenFinder/) [36]. The presence of antibiotic resistance genes was analyzed using a web-based server of Resistance gene identifier (RGI) v5.1.1 module of Comprehensive Antibiotic Resistance Database (CARD) v3.1.1 by including loose hits (https://card.mcmaster.ca/) [37].

## Supplementary Information

**Additional file 1.**

## Data Availability

All the data are available in appropriate web portals. 16S rRNA gene sequence data of *G. arilaitensis* LJH19 is available in NCBI GenBank with accession no. MT349443 (https://www.ncbi.nlm.nih.gov/nuccore/MT349443.1/). The whole-genome sequence data of *G. arilaitensis* LJH19 is available in NCBI GenBank with accession no. SPDS00000000 (https://www.ncbi.nlm.nih.gov/nuccore/SPDS00000000).
