# Correction to: Comparative genomics and physiological investigation supported safety, cold adaptation, efficient hydrolytic and plant growth-promoting potential of psychrotrophic *Glutamicibacter arilaitensis* LJH19, isolated from night-soil compost

**DOI:** 10.1186/s12864-021-07681-4

**Published:** 2021-05-18

**Authors:** Shruti Sinai Borker, Aman Thakur, Sanjeet Kumar, Sareeka Kumari, Rakshak Kumar, Sanjay Kumar

**Affiliations:** 1 Biotechnology Division, CSIR-Institute of Himalayan Bioresource Technology Palampur, Palampur, Himachal Pradesh 176061 India; 2 Academy of Scientific and Innovative Research (AcSIR), CSIR- Human Resource Development Centre, Ghaziabad, Uttar Pradesh 201 002 India

**Correction to:** *BMC Genomics* **22,** 307 (2021)

https://doi.org/10.1186/s12864-021-07632-z

Following publication of the original article [1], it was reported that there was an error in Fig. 4. The originally published Fig. 4 was missing the following five enzymes: Argininosuccinate lyase (argH), Arginine decarboxylase (speC), Agmatinase (speB), Polyamine aminopropyl transferase (speE), Ornithine decarboxylase (speC).

**Fig. 4:**
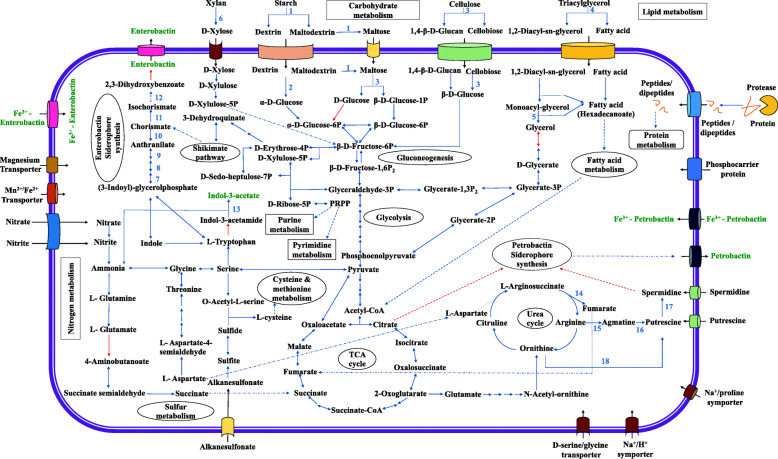
Schematic representation of the predicted genes encoding catabolic activities, transport and plant-growth promotion in *G. arilaitensis* LJH19. The selected key genes involved in the pathway indicated by blue arrows are: 1, amylase; 2, Oligo-1,6-glucosidase; 3, Beta-glucosidase; 4, Triacylglyceride lipase; 5, monoacylglycerol lipase; 6, Anthranilate synthase component I (TrpE); 7, Anthranilate phosphoribosyl transferase (TrpD); 7,Phosphoribosyl anthranilate isomerase (TrpF); 9, Indole-3-glycerol phosphate synthase (TrpC); 11, Isochorismate synthase (menF); 12, Isochorismatase; 13, Amidase; 14, Argininosuccinate lyase (argH); 15, Arginine decarboxylase (speC); 16, Agmatinase (speB); 17, Polyamine aminopropyl transferase (speE); 18, Ornithine decarboxylase (speC). Core metabolic enzymes indicated in the pathway by blue arrows are listed in supplementary Table S2. Red arrows indicate enzymes missing in the metabolic pathway. Multistep pathways are denoted with dotted lines

The correct figure is included in this Correction article and the original article has been updated.

## References

[1] Borker SS, Thakur A, Kumar S, Kumari S, Kumar R, Kumar S (2021). Comparative genomics and physiological investigation supported safety, cold adaptation, efficient hydrolytic and plant growth-promoting potential of psychrotrophic *Glutamicibacter arilaitensis* LJH19, isolated from night-soil compost. BMC Genomics.

