# Supplementary material for: Comparative genomics and physiological investigation supported safety, cold adaptation, efficient hydrolytic and plant growth-promoting potential of psychrotrophic Glutamicibacter arilaitensis LJH19, isolated from night-soil compost
Source: BMC Genomics. 2021 Apr 28;22:307. doi: 10.1186/s12864-021-07632-z (PMC8082909; doi:10.1186/s12864-021-07632-z)
Supplement: Supplementary file 1 — Additional file 1. [file 12864_2021_7632_MOESM1_ESM.pdf]

Comparative genomics and physiological investigation supported safety, cold adaptation, efficient hydrolytic and plant growth promoting potential of psychrotrophic *Glutamicibacter arilaitensis* LJH19, isolated from night soil compost.

Shruti Sinai Borker, Aman Thakur, Sanjeet Kumar, Sareeka Kumari, Rakshak Kumar*, Sanjay Kumar

Supplementary Figure S1

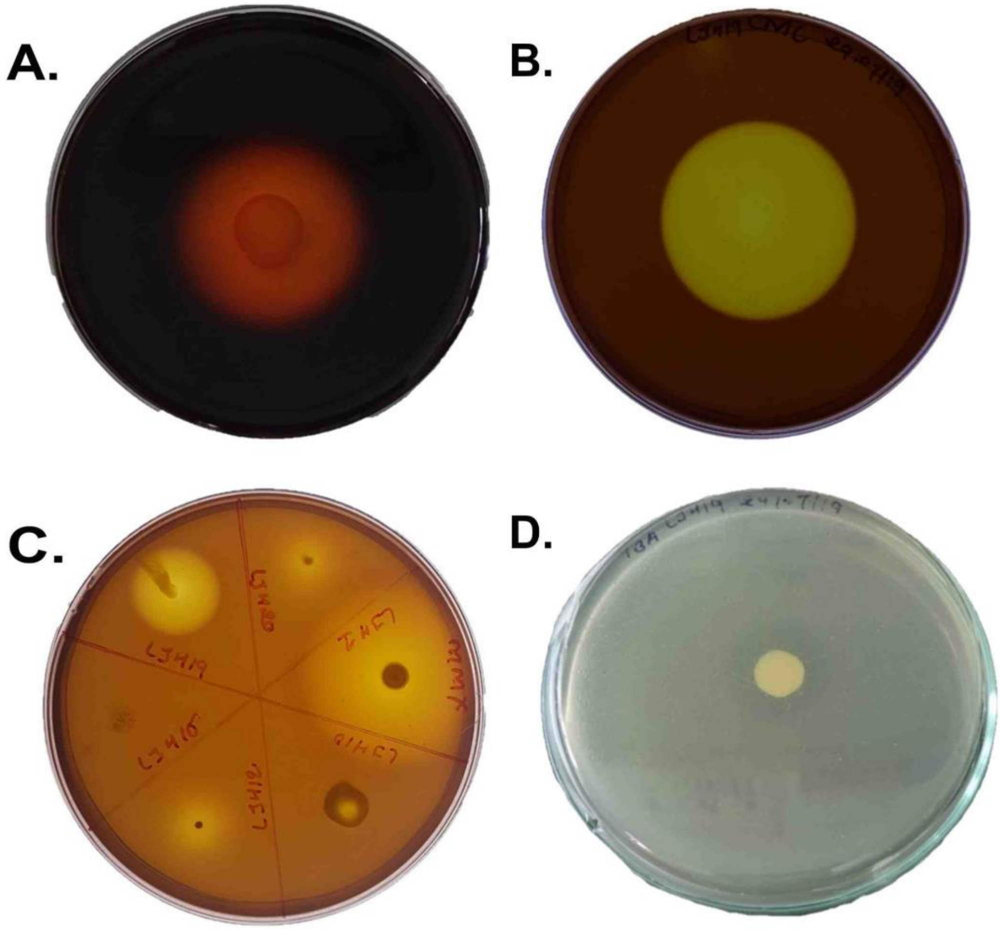

Fig. S1. Plate assay for qualitative estimation of enzymatic activity by LJH19. A) Amylase, B) Cellulase, C) Xylanase, D) Lipase. Clear halo zones around the colony indicated positive results.

Supplementary Figure S2

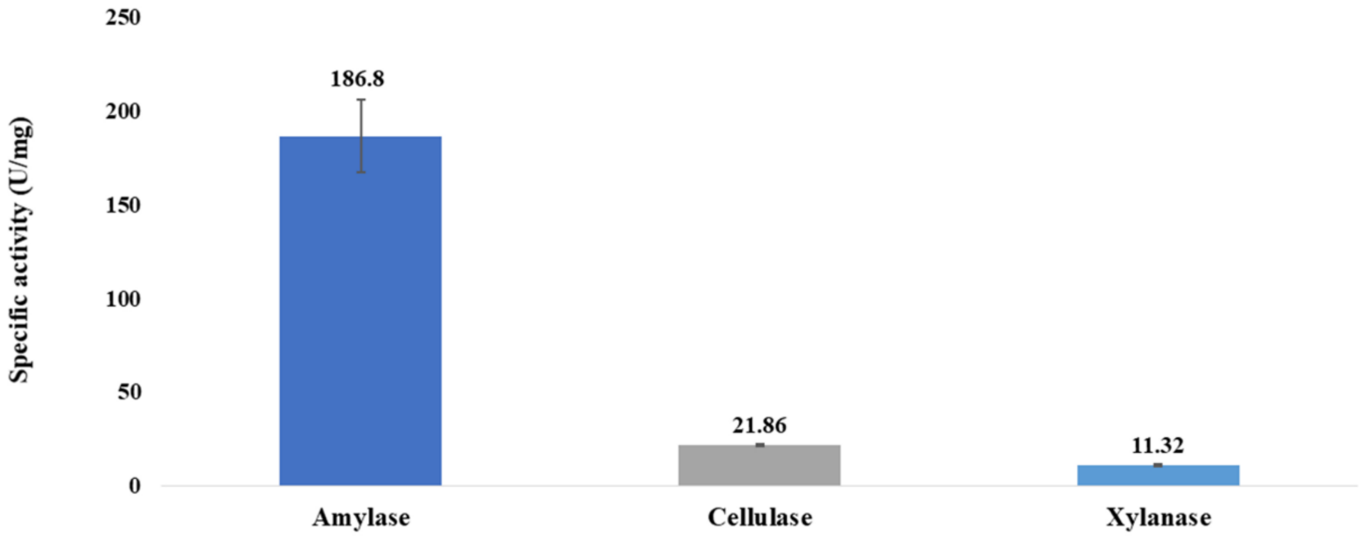

Fig. S2. Quantitative estimation of hydrolytic enzymes by LJH19. The specific activity of amylase, 186.76 ± 19.28 U/mg; cellulase, 21.85 ± 0.7 U/mg; and xylanase, 11.31± 0.51 U/mg at 15°C.

Supplementary Figure S3

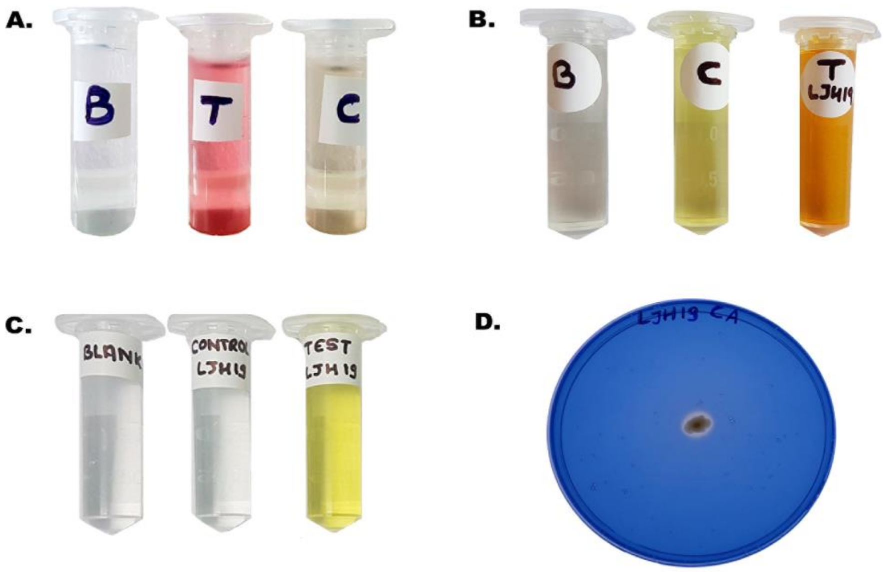

Fig. S3. Quantitative and qualitative estimation of Plant Growth Promoting traits by LJH19. A) Auxin Production, B) Ammonia production, C) Phosphate solubilization, D) Siderophore production on CAS Agar.

B-Blank, T-Test, C-Control

Supplementary Figure S4

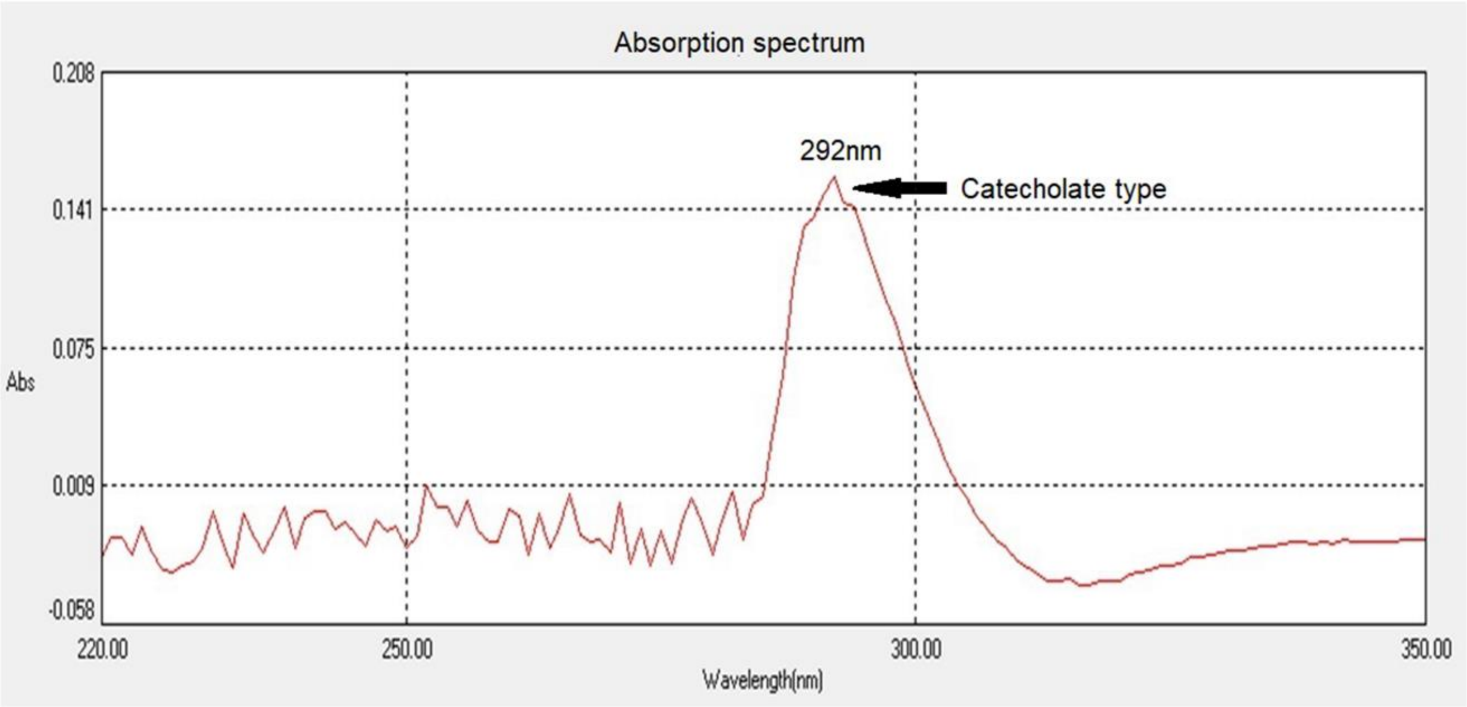

Fig. S4. Absorption spectra of cell-free supernatant of *G. arilaitensis* LJH19. A peak at 292 nm was observed in the absorption spectra indicating the presence of 2,3-dihydroxybenzoic acid (DHB), an intermediate of catecholate type siderophore.

Supplementary Figure S5

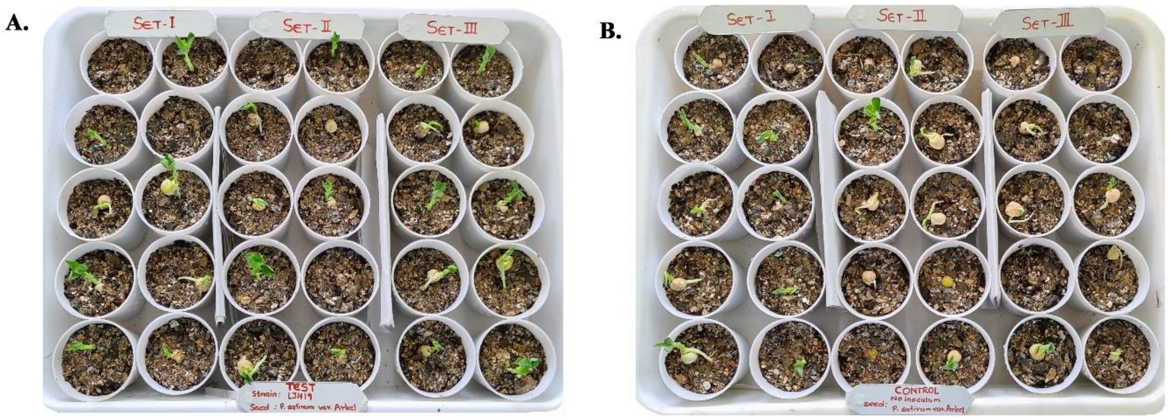

Fig. S5. Effect of plant growth promoting strain *Glutamicibacter arilaitensis* LJH19 treatments on germination of pea seeds (*Pisum sativum* var. Arkel). A) Test: pea seeds inoculated with LJH19 strain. **B)** Control: inoculated with sterile deionised water. The experiment was carried out in three sets with 10 replicates each.

Supplementary Figure S6

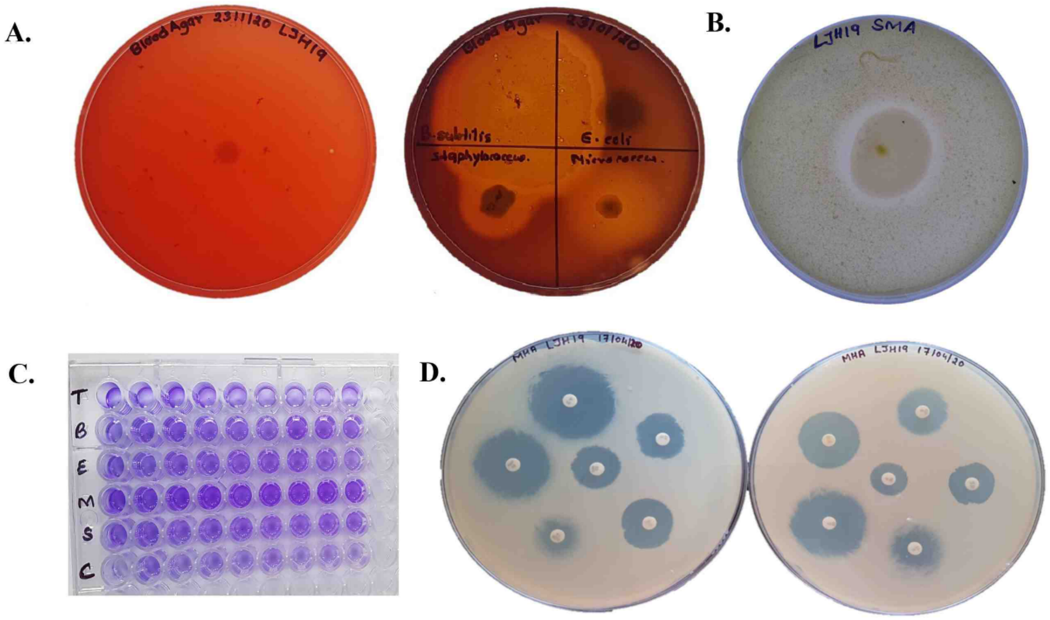

Fig. S6. Assessment of pathogenic potential of LJH19. A) Haemolytic activity; no clear zones around the colony indicated no haemolytic activity by LJH19, in comparison to haemolytic strains which showed beta haemolysis (clear zone) and alpha haemolysis (green or brown discoloration in the medium). B) Qualitative estimation of protease activity. Clear zone around the colony indicated positive results. C) Quantitative estimation of biofilm formation. T-Test, B-*Bacillus subtilis* (MTCC 121), E-*Escherichia coli* (MTCC 43), M- *Micrococcus luteus* (MTCC 2470), S-*Staphylococcus aureus subsp. aureus* (MTCC 96) , C- Control. D) Antibiotic susceptibility test. LJH19 was susceptible to all the twelve antibiotics tested i.e15 mcg, Azithromycin (AZM); 10 mcg, Ampicillin (AMP); 5 mcg, Ciprofloxacin (CIP); 30 mcg, Chloramphenicol (CHL); 15 mcg Erythromycin (E); 10 mcg, Gentamycin (G); 30 mcg, Kanamycin (K); 10 Units, Penicillin-G (P); 5 mcg, Rifampicin (RIF); 10 mcg, Streptomycin (S); 30 mcg, Tetracycline (TE); 30 mcg, Vancomycin (VA).

Supplementary Table S1. Genome features of all the strain of *Glutamicibacter* sp. and strain *G. arilaitensis* LJH19 with its geographical attributes.

Genome Name	Genome Status	Coverage (x)	# Contigs	Genome Length (bps)	% GC Content	# RefSeq CDS	Isolation source	%Completeness/ %Contamination	Accession No.
Glutamicibacter arilaitensis LJH19	WGS	153.0x	4	3602821	59.60	3245	Night soil compost	99.65/1.38	NZ_SPDS00000000
Glutamicibacter arilaitensis Re117	Complete	-	2	3909664	59.28	3423	Cheese	99.74/0.46	NC_014550
Glutamicibacter soli M275	WGS	500.0x	319	3965931	64.09	3800	-	99.28/2.14	NZ_WYDN00000000
Glutamicibacter soli NHPC-3	WGS	100.0x	25	3840670	64.34	3495	Tea plant rhizospheric soil	99.28/0.69	NZ_POAF00000000
Glutamicibacter uratoxydans NBRC 15515	WGS	152.0x	49	3786014	61.09	3533	-	99.74/0.58	NZ_BJNY00000000
Glutamicibacter nicotianae NBRC 14234	WGS	162.0x	43	3554887	61.92	3311	-	99.74/1.19	NZ_BJNE00000000
Glutamicibacter nicotianae OTC-	Complete	380.0x	3	3797724	61.72	-	Active sludge around	99.28/1.53	NZ_CP033081

16							pharmaceutical factory		
Glutamicibacter sp. JC586	WGS	100.0x	28	3524842	55.57	-	Soil	99.51/1.49	NZ_VHIN00000000
Glutamicibacter mysorens DSM 12798	WGS	34.0x	1	3459735	61.95	3177	-	99.74/0.96	NZ_PGEY00000000
Glutamicibacter arilaitensis JB182	WGS	50.0x	12	3947886	59.16	3708	Cheese rind	99.74/0.54	NZ_PNQX00000000
Glutamicibacter sp. HZAU	WGS	100.0x	40	3508313	61.70	3212	Bto:0003809	99.68/0.38	NZ_SCKZ00000000
Glutamicibacter sp. V16R2B1	WGS	12.0x	86	3479500	65.68	3197	Date palm rhizosphere	99.05/1.15	NZ_VATX00000000
Glutamicibacter sp. BW80	WGS	50.0x	78	4049569	60.38	3720	Cheese rind	99.74/0.61	NZ_NRGV00000000
Glutamicibacter sp. BW78	WGS	50.0x	43	3419483	64.04	3133	Cheese rind	99.77/0.61	NZ_NRGU00000000
Glutamicibacter sp. BW77	WGS	50.0x	90	3902028	56.57	3605	Cheese rind	99.51/0.5	NZ_NRGT00000000
Glutamicibacter halophytocola DR408	Complete	231.0x	1	3770186	60.19	3384	Rhizosphere of Soybean	99.51/1.15	NZ_CP042260

Glutamicibacter sp. 0426	WGS	241.0x	23	3549469	62	-	Soil	99.74/0.8	NZ_MPBI00000000
Glutamicibacter creatinolyticus LGCM 259	Complete	286.0x	1	3309128	65.55	2882	Abcess of a mare	99.51/0.69	NZ_CP034412
Glutamicibacter mysorens NBRC 103060	WGS	130.0x	15	3427456	62.02	-	-	99.74/0.96	NZ_BCQO00000000
Glutamicibacter sp. ZJUTW	Complete	100.0x	2	3673306	61.79	3379	Activated sludge	99.74/0.8	NZ_CP043624
Glutamicibacter halophytocola KLBMP 5180	Complete	100.0x	2	3,918,424	60.0	3545	Inner tissues of halophyte Limonium sinense (Girard) Kuntze	99.50/1.03	NZ_CP012750
Glutamicibacter mishrai S5-52	Complete	700.0x	1	3570747	59.44	3238	Coral mucus	99.50/1.94	NZ_CP032549

Supplementary Table S2. List of unique genes involved in catabolic activity, transport, and PGP activity retrieved from retrieved from pan genome analysis for G. arilaitensis LJH19 genome.

Locus tag	Gene	EC number	COG	Product
**Catabolic activity**				
MJJNNIGE_00069	pepO	3.4.24.-	COG3590	Neutral endopeptidase
MJJNNIGE_00216		3.1.2.-	COG2050	Putative esterase
MJJNNIGE_00245	lacZ	3.2.1.23		Beta-galactosidase
MJJNNIGE_00546		3.2.1.-		Beta-xylosidase
MJJNNIGE_01053	pulA	3.2.1.41		Pullulanase
MJJNNIGE_01084		3.4.21.-	COG0265	Serine protease
MJJNNIGE_01136	aml	3.2.1.1		Alpha-amylase
MJJNNIGE_01285	sacC	3.2.1.80	COG1621	Levanase
MJJNNIGE_01544, MJJNNIGE_01174	htpX	3.4.24.-		Protease HtpX
MJJNNIGE_02347	malL	3.2.1.10	COG0366	Oligo-1,6-glucosidase
MJJNNIGE_02452		3.1.1.23		Thermostable monoacylglycerol lipase
MJJNNIGE_02932	rip1	3.4.24.-	COG0750	Zinc metalloprotease Rip1
MJJNNIGE_03107	caeB	3.1.1.-		Carboxylesterase B
MJJNNIGE_03134	bglA	3.2.1.21	COG2723	Beta-glucosidase A
MJJNNIGE_03364	xylA	5.3.1.5		Xylose isomerase
MJJNNIGE_03385		3.2.1.-		Beta-xylosidase
**Plant-growth promoting activity**				
MJJNNIGE_00110	amiB2	3.5.1.4	COG0154	Putative amidase AmiB2
MJJNNIGE_00146	trpG	4.1.3.27	COG0512	Anthranilate synthase component 2
MJJNNIGE_00383	hpaH	1.14.14.8	COG2368	Anthranilate 3-monooxygenase oxygenase component
MJJNNIGE_00674, MJJNNIGE_00678	nasD	1.7.1.4	COG1251	Nitrite reductase [NAD(P)H]
MJJNNIGE_00675				hypothetical protein
MJJNNIGE_00679	nasC	1.7.-.-	COG0243	Assimilatory nitrate reductase catalytic subunit

MJJNNIGE_00796, MJJNNIGE_00881	andAa	1.18.1.3		Anthranilate 1,2-dioxygenase system ferredoxin--NAD(+) reductase component
MJJNNIGE_00805	puo	1.4.3.10		Putrescine oxidase
MJJNNIGE_01047, MJJNNIGE_01165	menF	5.4.4.2		Isochorismate synthase MenF
MJJNNIGE_01620	trpE	4.1.3.27	COG0147	Anthranilate synthase component 1
MJJNNIGE_01728	sir	1.8.7.1	COG0155	Sulfite reductase [ferredoxin]
MJJNNIGE_01926	trpD	2.4.2.18	COG0547	Anthranilate phosphoribosyltransferase
MJJNNIGE_02216, MJJNNIGE_03410	speE	2.5.1.16		Polyamine aminopropyltransferase (Spermidine synthase)
MJJNNIGE_03196	yecD	3.-.-.-	COG1335	Isochorismatase family protein YecD
MJJNNIGE_03076	argD	2.6.1.11	COG4992	Acetylornithine aminotransferase
MJJNNIGE_03077	argF	2.1.3.3	COG0078	Ornithine carbamoyltransferase
MJJNNIGE_03081	argH	4.3.2.1	COG0165	Argininosuccinate lyase
MJJNNIGE_00820	hutG	3.5.3.8	COG0010	Formimidoylglutamase* (Arginase)
MJJNNIGE_00897	speC			hypothetical protein*(ornithine decarboxylase)
MJJNNIGE_02562	speB			Guanidinobutyrase (agmatinase)
MJJNNIGE_02361		3.6.1.66	COG0127	dITP/XTP pyrophosphatase
MJJNNIGE_02823	yieH	3.1.3.-	COG0637	6-phosphogluconate phosphatase
MJJNNIGE_00097	ppa	3.6.1.1	COG0221	Inorganic pyrophosphatase
MJJNNIGE_00105	ywpJ	3.1.3.-	COG0561	Phosphatase YwpJ
MJJNNIGE_02565	suhB	3.1.3.25	COG0483	Inositol-1-monophosphatase
**Transporters**				
MJJNNIGE_00015, MJJNNIGE_00589, MJJNNIGE_00968,	yhdG		COG0531	putative amino acid permease YhdG

MJJNNIGE_00049	pstB1	7.3.2.1	COG1117	Phosphate import ATP-binding protein PstB 1
MJJNNIGE_00050				hypothetical protein
MJJNNIGE_00051	pstC2		COG0573	Phosphate transport system permease protein PstC 2
MJJNNIGE_00122	xylE			D-xylose-proton symporter
MJJNNIGE_00111	dtpT		COG3104	Di-/tripeptide transporter
MJJNNIGE_00242	xylG	7.5.2.10	COG1129	Xylose import ATP-binding protein XylG
MJJNNIGE_00243	xylH		COG4214	Xylose transport system permease protein XylH
MJJNNIGE_00373, MJJNNIGE_00374, MJJNNIGE_03335	ssuC		COG0600	Putative aliphatic sulfonates transport permease protein SsuC
MJJNNIGE_00413	fatE	7.2.2.-	COG4604	Petrobactin import ATP-binding protein FatE
MJJNNIGE_00414	fatC		COG4605	Petrobactin import system permease protein FatC
MJJNNIGE_00415	fatD		COG4606	Petrobactin import system permease protein FatD
MJJNNIGE_00416	yclQ		COG4607	Petrobactin-binding protein YclQ
MJJNNIGE_01051, MJJNNIGE_01739	apeX		COG2409	Apo-petrobactin exporter
MJJNNIGE_00510	puuP		COG0531	Putrescine importer PuuP
MJJNNIGE_00547, MJJNNIGE_00784, MJJNNIGE_03386	ngcG		COG0395	Diacetylchitobiose uptake system permease protein NgcG
MJJNNIGE_00548	ngcF		COG1175	Diacetylchitobiose uptake system permease protein NgcF
MJJNNIGE_00551			COG1132	putative ABC transporter ATP-binding protein
MJJNNIGE_00552		7.6.2.-	COG1132	Fatty acid ABC transporter ATP-binding/permease protein

MJJNNIGE_00597	cycA		COG1113	D-serine/D-alanine/glycine transporter
MJJNNIGE_00680	narK		COG2223	Nitrate/nitrite transporter NarK
MJJNNIGE_00779	gntT		COG2610	High-affinity gluconate transporter
MJJNNIGE_00959	yfiZ		COG0609	putative siderophore transport system permease protein YfiZ
MJJNNIGE_00960	yfhA		COG0609	putative siderophore transport system permease protein YfhA
MJJNNIGE_01002	potD		COG0687	Spermidine/putrescine-binding periplasmic protein
MJJNNIGE_01004	potA	7.6.2.11	COG3842	Spermidine/putrescine import ATP-binding protein PotA
MJJNNIGE_01022, MJJNNIGE_02404, MJJNNIGE_02770, MJJNNIGE_03351	entS			Enterobactin exporter EntS
MJJNNIGE_01290, MJJNNIGE_01459, MJJNNIGE_02914,	dppB		COG0601	Dipeptide transport system permease protein DppB
MJJNNIGE_01291	dppC		COG1173	Dipeptide transport system permease protein DppC
MJJNNIGE_01292	dppD		COG0444	Dipeptide transport ATP-binding protein DppD
MJJNNIGE_01293	oppF		COG4608	Oligopeptide transport ATP-binding protein OppF
MJJNNIGE_01734	nrtB		COG0600	Nitrate import permease protein NrtB
MJJNNIGE_02149	yfhA_2		COG0609	putative siderophore transport system permease protein YfhA
MJJNNIGE_02150	yfiZ_2		COG0609	putative siderophore transport system permease protein YfiZ

MJJNNIGE_02151	yfiY		COG0614	putative siderophore-binding lipoprotein YfiY
MJJNNIGE_02344	malG		COG3833	Maltose/maltodextrin transport system permease protein MalG
MJJNNIGE_02345	malF		COG1175	Maltose/maltodextrin transport system permease protein MalF
MJJNNIGE_02346	cycB		COG2182	Cyclodextrin-binding protein
MJJNNIGE_02833	melC		COG0395	Melibiose/raffinose/stachyose import permease protein MelC
MJJNNIGE_02856	fepC		COG1120	Ferric enterobactin transport ATP-binding protein FepC
MJJNNIGE_02857	fepG		COG4779	Ferric enterobactin transport system permease protein FepG
MJJNNIGE_02858	fepD		COG0609	Ferric enterobactin transport system permease protein FepD
MJJNNIGE_02913	oppA		COG4166	Oligopeptide-binding protein OppA
MJJNNIGE_02915	oppC		COG1173	Oligopeptide transport system permease protein OppC
MJJNNIGE_02920			COG1173	Putative peptide transport permease protein
MJJNNIGE_03304	fepB		COG4592	Ferrienterobactin-binding periplasmic protein
MJJNNIGE_03333	nrtD	7.3.2.4	COG1116	Nitrate import ATP-binding protein NrtD
MJJNNIGE_03387	melD		COG1175	Melibiose/raffinose/stachyose import permease protein MelD

Supplementary Table S3. Virulence factors annotated in *G. arilaitensis* LJH19 genome.

Source ID	Gene	Product	Source organism	Length	E-value	Overall identity	Gaps	Query aligned	Subject aligned
VFG015784	gacA	two component transcriptional regulator	*Pseudomonas putida* GB-1	546	0.003	96.7% (29/30)	0	200302-200331	136-165
VFG016067	pvdL	peptide synthase (Pyoverdine)	*Pseudomonas fluorescens* Pf-5	13029	7e-04	90.7% (39/43)	0	213705-213747	7062-7020
VFG016123	pchE	pyochelin synthetase E (Pyochelin)	*Pseudomonas fluorescens* Pf-5	3471	0.003	94.1% (32/34)	0	213705-213738	2157-2124
VFG029722	mps2	linear gramicidin synthetase subunit B	*Mycobacterium avium* 104	7659	4e-05	93.4% (71/76)	0	213705-213741, 213709-213747	603-567, 5075-5037
VFG029725	mps2	non-ribosomal peptide synthetase	*Mycobacterium marinum* M	11670	0.003	90.5% (114/126)	0	213705-213746, 213705-213746, 213705-213746	1905-1864, 5106-5065, 9663-9622
VFG030388	fadD13	Probable chain-fatty-acid-CoA ligase FadD13 (fatty-acyl-CoA synthetase)	*Mycobacterium tuberculosis* H37Rv	1512	0.003	96.7% (29/30)	0	212970-212999	1224-1195
VFG030389	fadD13	substrate--CoA ligase (MymA operon)	*Mycobacterium tuberculosis* CDC1551	1512	0.003	96.7% (29/30)	0	212970-212999	1224-1195
VFG030391	fadD13	FadD13 (MymA operon)	*Mycobacterium avium* subsp. *paratuberculosis* K-10	1533	0.003	92.1% (35/38)	0	213704-213741	526-489
VFG030392	fadD13	acyl-CoA dehydrogenase	*Mycobacterium avium* 104	1533	0.003	92.1% (35/38)	0	213704-213741	526-489
VFG030398	fadD13	putative chain-fatty-acid-CoA ligase	*Mycobacterium canettii* CIPT 140010059	1512	0.003	96.7% (29/30)	0	212970-212999	1224-1195

VFG030404	fadD13	Putative chain-fatty-acid-CoA ligase FadD13 (fatty-acyl-CoA synthetase) [MymA operon]	*Mycobacterium canettii* CIPT 140060008	1512	0.003	96.7% (29/30)	0	212970-212999	1224-1195
VFG030405	fadD13	Putative chain-fatty-acid-CoA ligase FadD13 (fatty-acyl-CoA synthetase) [MymA operon]	*Mycobacterium canettii* CIPT 140070008	1512	0.003	96.7% (29/30)	0	212970-212999	1224-1195
VFG030406	fadD13	Putative chain-fatty-acid-CoA ligase FadD13 (fatty-acyl-CoA synthetase) [MymA operon]	*Mycobacterium canettii* CIPT 140070010	1512	0.003	96.7% (29/30)	0	212970-212999	1224-1195
VFG030407	fadD13	Putative chain-fatty-acid-CoA ligase FadD13 (fatty-acyl-CoA synthetase) [MymA operon]	*Mycobacterium canettii* CIPT 140070017	1512	0.003	96.7% (29/30)	0	212970-212999	1224-1195
VFG030411	fadD13	chain-fatty-acid-CoA ligase [MymA operon]	*Mycobacterium tuberculosis* str. Beijing/NITR203	1512	0.003	96.7% (29/30)	0	212970-212999	1224-1195
VFG030416	fadD13	chain-fatty-acid-CoA ligase [MymA operon]	*Mycobacterium tuberculosis* str. Haarlem/NITR202	990	0.003	96.7% (29/30)	0	212970-212999	645-616
VFG030419	fadD13	putative chain-fatty-acid-coa ligase fadd13 (fatty-acyl-coa synthetase) [MymA operon]	*Mycobacterium tuberculosis* 7199-99	1512	0.003	96.7% (29/30)	0	212970-212999	1224-1195
VFG031042	zmp1	metallopeptidase [Zn++ metallophrotease]	*Mycobacterium smegmatis* str. MC2 155	2001	7e-04	88.2% (45/51)	0	64981-65031	1713-1663
VFG031043	zmp1	PgPepO oligopeptidase [Zn++ metallophrotease]	*Mycobacterium* sp. JLS	1995	3e-06	87.3% (55/63)	0	65164-65226	1506-1444
VFG031050	zmp1	Probable zinc metalloprotease [Zn++ metallophrotease]	*Mycobacterium abscessus* ATCC 19977	1983	7e-04	83.1% (69/83)	0	65120-65202	1556-1474

VFG031064	zmp1	metalloendopeptidase PepO [Zn++ metallophrotease]	*Mycobacterium abscessus* subsp. bolletii 50594	1983	7e-04	83.1% (69/83)	0	65120-65202	1556-1474
VFG031068	zmp1	Neutral endopeptidase [Zn++ metallophrotease]	*Mycobacterium abscessus* subsp. bolletii str. GO 06	1983	7e-04	83.1% (69/83)	0	65120-65202	1556-1474
VFG031151	Mpa	vesicle-fusing ATPase [Proteasome-associated proteins	*Mycobacterium vanbaalenii* PYR-1	1848	7e-04	86.4% (51/59)	0	90318-90376	932-874
VFG035575	aec27/clpV	putative type VI secretion system protein [ACE T6SS]	*Escherichia coli* O44:H18 042	2733	0.003	87.0% (47/54)	0	60323-60376	2078-2025

Supplementary Table S4. The pathogenic potential of strain *G. arilaitensis* LJH19 assessed using the PathogenFinder 1.1 (https://cge.cbs.dtu.dk/services/PathogenFinder/). The predicted results identified LJH19 strain as a non-human pathogen.

Min Identity Threshold	Z-Threshold	Prediction Score	Probability of being human pathogen	Matches	Genome Coverage (%)	Pathogenic Families Matched	Non-Pathogenic Families Matched	The organism is predicted as human pathogenic
83.995	3.0	-3.451	0.356	5	0.15	1	4	No

Supplementary Table S5. Antibiotic resistance genes identified in *G. arilaitensis* LJH19 genome.

Contig	Start	Stop	Orientation	Best hit ARO	Drug class	AMR gene family
SPDS01000001.1_18	23219	23818	+	*Escherichia coli* acrR with mutation conferring multidrug antibiotic resistance	fluoroquinolone antibiotic; cephalosporin; glycylcycline; penam; tetracycline antibiotic; rifamycin antibiotic; phenicol antibiotic; triclosan	resistance-nodulation-cell division (RND) antibiotic efflux pump
SPDS01000001.1_20	24429	25814	+	THIN-B	carbapenem; cephalosporin; penam	THIN-B beta-lactamase
SPDS01000001.1_34	35896	36597	+	Erm(37)	macrolide antibiotic; lincosamide antibiotic; streptogramin antibiotic	Erm 23S ribosomal RNA methyltransferase
SPDS01000001.1_36	37755	39362	+	lmrC	macrolide antibiotic; lincosamide antibiotic; streptogramin antibiotic; tetracycline antibiotic; oxazolidinone antibiotic; phenicol antibiotic; pleuromutilin antibiotic	ABC-F ATP-binding cassette ribosomal protection protein
SPDS01000001.1_45	45306	46085	-	RanA	aminoglycoside antibiotic	ATP-binding cassette (ABC) antibiotic efflux pump
SPDS01000001.1_57	57720	58118	+	ramA	fluoroquinolone antibiotic; monobactam; carbapenem; cephalosporin; glycylcycline; cephamycin; penam; tetracycline antibiotic; rifamycin antibiotic; phenicol antibiotic; triclosan; penem	resistance-nodulation-cell division (RND) antibiotic efflux pump; General Bacterial Porin with reduced permeability to beta-lactams
SPDS01000001.1_80	87549	88451	-	sul4	sulfonamide antibiotic	sulfonamide resistant sul
SPDS01000001.1_112	118845	120137	+	bcr-1	bicyclomycin	major facilitator superfamily (MFS) antibiotic efflux pump
SPDS01000001.1_132	138433	139896	+	*Haemophilus influenzae* PBP3 conferring resistance to beta-lactam antibiotics	cephalosporin; cephamycin; penam	Penicillin-binding protein mutations conferring resistance to beta-lactam antibiotics
SPDS01000001.1_139	145763	146161	-	qacE		major facilitator superfamily (MFS) antibiotic efflux pump
SPDS01000001.1_175	190226	190576	-	PmrF	peptide antibiotic	pmr phosphoethanolamine transferase
SPDS01000001.1_182	197049	197975	+	bcrA	peptide antibiotic	ATP-binding cassette (ABC) antibiotic efflux pump
SPDS01000001.1_185	200152	200856	+	evgA	macrolide antibiotic; fluoroquinolone antibiotic; penam; tetracycline antibiotic	major facilitator superfamily (MFS) antibiotic efflux pump; resistance-nodulation-cell division (RND) antibiotic efflux pump
SPDS01000001.1_204	218207	219589	+	otr(B)	tetracycline antibiotic	major facilitator superfamily (MFS) antibiotic efflux pump
SPDS01000001.1_207	221061	221522	+	mgrA	fluoroquinolone antibiotic; cephalosporin; penam; tetracycline antibiotic; peptide antibiotic; acridine dye	ATP-binding cassette (ABC) antibiotic efflux pump; major facilitator superfamily (MFS) antibiotic efflux pump

SPDS01000001.1_225	236342	237880	+	bcrA	peptide antibiotic	ATP-binding cassette (ABC) antibiotic efflux pump
SPDS01000001.1_242	256565	258418	+	novA	aminocoumarin antibiotic	ATP-binding cassette (ABC) antibiotic efflux pump
SPDS01000001.1_264	280764	281351	-	*Escherichia coli* acrR with mutation conferring multidrug antibiotic resistance	fluoroquinolone antibiotic; cephalosporin; glycylcycline; penam; tetracycline antibiotic; rifamycin antibiotic; phenicol antibiotic; triclosan	resistance-nodulation-cell division (RND) antibiotic efflux pump
SPDS01000001.1_272	287804	289270	+	Staphylococcus aureus LmrS	macrolide antibiotic; aminoglycoside antibiotic; oxazolidinone antibiotic; diaminopyrimidine antibiotic; phenicol antibiotic	major facilitator superfamily (MFS) antibiotic efflux pump
SPDS01000001.1_273	289494	290957	+	fexA	phenicol antibiotic	major facilitator superfamily (MFS) antibiotic efflux pump
SPDS01000001.1_278	295468	296904	-	fexA	phenicol antibiotic	major facilitator superfamily (MFS) antibiotic efflux pump
SPDS01000001.1_294	310386	311864	-	fexA	phenicol antibiotic	major facilitator superfamily (MFS) antibiotic efflux pump
SPDS01000001.1_301	317459	318760	+	MexL	macrolide antibiotic; tetracycline antibiotic; triclosan	resistance-nodulation-cell division (RND) antibiotic efflux pump
SPDS01000001.1_311	327167	328474	+	norB	fluoroquinolone antibiotic	major facilitator superfamily (MFS) antibiotic efflux pump
SPDS01000001.1_326	344729	346060	+	*Acinetobacter baumannii* AbaF	fosfomycin	major facilitator superfamily (MFS) antibiotic efflux pump
SPDS01000001.1_333	353816	354742	-	NmcR	carbapenem; cephalosporin; cephamycin; penam	NmcA beta-lactamase
SPDS01000001.1_344	363794	365422	-	iri	rifamycin antibiotic	rifampin monooxygenase
SPDS01000001.1_355	375789	377453	+	iri	rifamycin antibiotic	rifampin monooxygenase
SPDS01000001.1_359	380234	381073	+	RanA	aminoglycoside antibiotic	ATP-binding cassette (ABC) antibiotic efflux pump
SPDS01000001.1_364	386866	387804	+	NmcR	carbapenem; cephalosporin; cephamycin; penam	NmcA beta-lactamase
SPDS01000001.1_366	389309	390730	-	*Acinetobacter baumannii* AbaF	fosfomycin	major facilitator superfamily (MFS) antibiotic efflux pump
SPDS01000001.1_374	398926	400065	-	RanA	aminoglycoside antibiotic	ATP-binding cassette (ABC) antibiotic efflux pump
SPDS01000001.1_387	410865	412037	+	*Staphylococcus aureus* norA	fluoroquinolone antibiotic	major facilitator superfamily (MFS) antibiotic efflux pump
SPDS01000001.1_391	413820	415868	-	vanO	glycopeptide antibiotic	glycopeptide resistance gene cluster; van ligase
SPDS01000001.1_397	421265	422020	-	msbA	nitroimidazole antibiotic	ATP-binding cassette (ABC) antibiotic efflux pump
SPDS01000001.1_407	430248	431327	+	rphA	rifamycin antibiotic	rifampin phosphotransferase

SPDS01000001.1_408	431385	433250	+	rphB	rifamycin antibiotic	rifampin phosphotransferase
SPDS01000001.1_414	438276	440159	+	iri	rifamycin antibiotic	rifampin monooxygenase
SPDS01000001.1_425	450901	452298	-	norB	fluoroquinolone antibiotic	major facilitator superfamily (MFS) antibiotic efflux pump
SPDS01000001.1_452	483818	485038	-	fexA	phenicol antibiotic	major facilitator superfamily (MFS) antibiotic efflux pump
SPDS01000001.1_453	485542	486057	+	AAC(6')-Ia	aminoglycoside antibiotic	AAC(6')
SPDS01000001.1_470	500397	501032	+	*Acinetobacter baumannii* AbaF	fosfomycin	major facilitator superfamily (MFS) antibiotic efflux pump
SPDS01000001.1_487	527795	529192	+	lmrC	macrolide antibiotic; lincosamide antibiotic; streptogramin antibiotic; tetracycline antibiotic; oxazolidinone antibiotic; phenicol antibiotic; pleuromutilin antibiotic	ABC-F ATP-binding cassette ribosomal protection protein
SPDS01000001.1_519	556557	558080	+	norB	fluoroquinolone antibiotic	major facilitator superfamily (MFS) antibiotic efflux pump
SPDS01000001.1_520	558113	559630	+	norB	fluoroquinolone antibiotic	major facilitator superfamily (MFS) antibiotic efflux pump
SPDS01000001.1_533	574212	575945	+	efrA	macrolide antibiotic; fluoroquinolone antibiotic; rifamycin antibiotic	ATP-binding cassette (ABC) antibiotic efflux pump
SPDS01000001.1_534	575946	578033	+	patB	fluoroquinolone antibiotic	ATP-binding cassette (ABC) antibiotic efflux pump
SPDS01000001.1_542	587897	589279	+	qacA	fluoroquinolone antibiotic	major facilitator superfamily (MFS) antibiotic efflux pump
SPDS01000001.1_545	591081	591686	+	*Escherichia coli* acrR with mutation conferring multidrug antibiotic resistance	fluoroquinolone antibiotic; cephalosporin; glycylcycline; penam; tetracycline antibiotic; rifamycin antibiotic; phenicol antibiotic; triclosan	resistance-nodulation-cell division (RND) antibiotic efflux pump
SPDS01000001.1_549	594823	596331	+	lmrB	lincosamide antibiotic	ATP-binding cassette (ABC) antibiotic efflux pump
SPDS01000001.1_559	604409	605704	+	vanYB	glycopeptide antibiotic	vanY; glycopeptide resistance gene cluster
SPDS01000001.1_562	607519	608718	+	qacB	fluoroquinolone antibiotic	major facilitator superfamily (MFS) antibiotic efflux pump
SPDS01000001.1_564	609784	611088	+	hmrM	fluoroquinolone antibiotic; acridine dye	multidrug and toxic compound extrusion (MATE) transporter
SPDS01000001.1_567	612821	614134	+	*Acinetobacter baumannii* AbaF	fosfomycin	major facilitator superfamily (MFS) antibiotic efflux pump
SPDS01000001.1_583	627822	628964	+	vanKI	glycopeptide antibiotic	glycopeptide resistance gene cluster; vanK
SPDS01000001.1_591	635588	637486	+	iri	rifamycin antibiotic	rifampin monooxygenase
SPDS01000001.1_614	656595	657713	+	tet(50)	tetracycline antibiotic	tetracycline inactivation enzyme

SPDS01000001.1_615	657850	659148	+	clbC	lincosamide antibiotic; streptogramin antibiotic; oxazolidinone antibiotic; phenicol antibiotic; pleuromutilin antibiotic	Cfr 23S ribosomal RNA methyltransferase
SPDS01000001.1_625	667327	668244	+	tetA(58)	tetracycline antibiotic	major facilitator superfamily (MFS) antibiotic efflux pump
SPDS01000001.1_628	670144	670749	+	vanRF	glycopeptide antibiotic	glycopeptide resistance gene cluster; vanR
SPDS01000001.1_699	748462	750057	-	bcrA	peptide antibiotic	ATP-binding cassette (ABC) antibiotic efflux pump
SPDS01000001.1_711	760233	761138	-	adeL	fluoroquinolone antibiotic; tetracycline antibiotic	resistance-nodulation-cell division (RND) antibiotic efflux pump
SPDS01000001.1_728	777457	778719	+	bcrA	peptide antibiotic	ATP-binding cassette (ABC) antibiotic efflux pump
SPDS01000001.1_734	782201	783379	-	bcr-1	bicyclomycin	major facilitator superfamily (MFS) antibiotic efflux pump
SPDS01000001.1_752	806706	807596	-	adeL	fluoroquinolone antibiotic; tetracycline antibiotic	resistance-nodulation-cell division (RND) antibiotic efflux pump
SPDS01000001.1_775	833507	834763	+	tet(42)	tetracycline antibiotic	major facilitator superfamily (MFS) antibiotic efflux pump
SPDS01000001.1_787	843002	844063	+	tet(31)	tetracycline antibiotic	major facilitator superfamily (MFS) antibiotic efflux pump
SPDS01000001.1_790	845073	845864	-	golS	monobactam; carbapenem; cephalosporin; cephamycin; penam; phenicol antibiotic; penem	resistance-nodulation-cell division (RND) antibiotic efflux pump
SPDS01000001.1_792	847244	848911	-	iri	rifamycin antibiotic	rifampin monooxygenase
SPDS01000001.1_800	854398	855153	+	oleC	macrolide antibiotic	ATP-binding cassette (ABC) antibiotic efflux pump
SPDS01000001.1_806	859241	860755	+	bcrA	peptide antibiotic	ATP-binding cassette (ABC) antibiotic efflux pump
SPDS01000001.1_814	866140	868596	+	macB	macrolide antibiotic	ATP-binding cassette (ABC) antibiotic efflux pump
SPDS01000001.1_833	885706	886806	-	vanRO	glycopeptide antibiotic	glycopeptide resistance gene cluster; vanR
SPDS01000001.1_836	888787	889665	-	vanJ	glycopeptide antibiotic	vanJ membrane protein
SPDS01000001.1_837	889874	891100	+	cmrA	phenicol antibiotic	major facilitator superfamily (MFS) antibiotic efflux pump
SPDS01000001.1_843	894394	895608	-	*Acinetobacter baumannii* AbaF	fosfomycin	major facilitator superfamily (MFS) antibiotic efflux pump
SPDS01000001.1_860	914530	915909	+	*Acinetobacter baumannii* AbaF	fosfomycin	major facilitator superfamily (MFS) antibiotic efflux pump
SPDS01000001.1_862	916698	917627	-	NmcR	carbapenem; cephalosporin; cephamycin; penam	NmcA beta-lactamase
SPDS01000001.1_874	929857	931086	-	ACT-22	carbapenem; cephalosporin; cephamycin; penam	ACT beta-lactamase

SPDS01000001.1_882	942765	943646	-	bcrA	peptide antibiotic	ATP-binding cassette (ABC) antibiotic efflux pump
SPDS01000001.1_883	943728	944393	-	evgA	macrolide antibiotic; fluoroquinolone antibiotic; penam; tetracycline antibiotic	major facilitator superfamily (MFS) antibiotic efflux pump; resistance-nodulation-cell division (RND) antibiotic efflux pump
SPDS01000001.1_888	948277	950016	+	tetA(46)	tetracycline antibiotic	ATP-binding cassette (ABC) antibiotic efflux pump
SPDS01000001.1_889	950016	951776	+	msbA	nitroimidazole antibiotic	ATP-binding cassette (ABC) antibiotic efflux pump
SPDS01000001.1_895	956793	958031	+	tet(39)	tetracycline antibiotic	major facilitator superfamily (MFS) antibiotic efflux pump
SPDS01000001.1_899	960139	961656	-	lfrA	fluoroquinolone antibiotic	major facilitator superfamily (MFS) antibiotic efflux pump
SPDS01000001.1_910	971683	972348	-	evgA	macrolide antibiotic; fluoroquinolone antibiotic; penam; tetracycline antibiotic	major facilitator superfamily (MFS) antibiotic efflux pump; resistance-nodulation-cell division (RND) antibiotic efflux pump
SPDS01000001.1_925	989003	990454	-	facT	elfamycin antibiotic	major facilitator superfamily (MFS) antibiotic efflux pump
SPDS01000001.1_930	994632	995375	+	macB	macrolide antibiotic	ATP-binding cassette (ABC) antibiotic efflux pump
SPDS01000001.1_933	997953	998753	-	SMB-1	carbapenem; cephalosporin; penam	SMB beta-lactamase
SPDS01000001.1_936	1000929	1002836	+	bcrA	peptide antibiotic	ATP-binding cassette (ABC) antibiotic efflux pump
SPDS01000001.1_938	1003443	1005272	+	novA	aminocoumarin antibiotic	ATP-binding cassette (ABC) antibiotic efflux pump
SPDS01000001.1_939	1005269	1005754	-	emrR	fluoroquinolone antibiotic	major facilitator superfamily (MFS) antibiotic efflux pump
SPDS01000001.1_964	1036808	1037218	-	arr-5	rifamycin antibiotic	rifampin ADP-ribosyltransferase (Arr)
SPDS01000001.1_979	1053663	1054796	+	tetA(58)	tetracycline antibiotic	major facilitator superfamily (MFS) antibiotic efflux pump
SPDS01000001.1_982	1056956	1057390	+	emrR	fluoroquinolone antibiotic	major facilitator superfamily (MFS) antibiotic efflux pump
SPDS01000001.1_997	1069344	1070600	+	mefC	macrolide antibiotic	major facilitator superfamily (MFS) antibiotic efflux pump
SPDS01000001.1_1001	1073699	1074463	-	macB	macrolide antibiotic	ATP-binding cassette (ABC) antibiotic efflux pump
SPDS01000001.1_1008	1080088	1080945	-	vanXYN	glycopeptide antibiotic	glycopeptide resistance gene cluster; vanXY
SPDS01000001.1_1018	1090836	1093163	+	novA	aminocoumarin antibiotic	ATP-binding cassette (ABC) antibiotic efflux pump
SPDS01000001.1_1024	1098991	1099479	-	emrR	fluoroquinolone antibiotic	major facilitator superfamily (MFS) antibiotic efflux pump

SPDS01000001.1_1049	1129359	1131167	-	novA	aminocoumarin antibiotic	ATP-binding cassette (ABC) antibiotic efflux pump
SPDS01000001.1_1050	1131164	1132987	-	novA	aminocoumarin antibiotic	ATP-binding cassette (ABC) antibiotic efflux pump
SPDS01000001.1_1055	1138274	1138951	-	CRP	macrolide antibiotic; fluoroquinolone antibiotic; penam	resistance-nodulation-cell division (RND) antibiotic efflux pump
SPDS01000001.1_1070	1153396	1154052	-	AAC(2')-Ia	aminoglycoside antibiotic	AAC(2')
SPDS01000001.1_1102	1183793	1185220	-	*Acinetobacter baumannii* AbaF	fosfomycin	major facilitator superfamily (MFS) antibiotic efflux pump
SPDS01000001.1_1111	1195627	1196244	-	*Escherichia coli* acrR with mutation conferring multidrug antibiotic resistance	fluoroquinolone antibiotic; cephalosporin; glycylcycline; penam; tetracycline antibiotic; rifamycin antibiotic; phenicol antibiotic; triclosan	resistance-nodulation-cell division (RND) antibiotic efflux pump
SPDS01000001.1_1117	1203027	1203782	+	RanA	aminoglycoside antibiotic	ATP-binding cassette (ABC) antibiotic efflux pump
SPDS01000001.1_1135	1221006	1221788	-	tetA(58)	tetracycline antibiotic	major facilitator superfamily (MFS) antibiotic efflux pump
SPDS01000001.1_1165	1252255	1252779	+	AAC(6')-Ib-cr9	fluoroquinolone antibiotic; aminoglycoside antibiotic	AAC(6')-Ib-cr
SPDS01000001.1_1168	1254339	1254914	-	dfrA26	diaminopyrimidine antibiotic	trimethoprim resistant dihydrofolate reductase dfr
SPDS01000001.1_1174	1260575	1261243	-	mtrA	macrolide antibiotic; penam	resistance-nodulation-cell division (RND) antibiotic efflux pump
SPDS01000001.1_1183	1268679	1269347	+	evgA	macrolide antibiotic; fluoroquinolone antibiotic; penam; tetracycline antibiotic	major facilitator superfamily (MFS) antibiotic efflux pump; resistance-nodulation-cell division (RND) antibiotic efflux pump
SPDS01000001.1_1193	1277520	1281026	+	*Mycobacterium tuberculosis* rpoB mutants conferring resistance to rifampicin	rifamycin antibiotic	rifamycin-resistant beta-subunit of RNA polymerase (rpoB)
SPDS01000001.1_1197	1286531	1288645	+	tetT	tetracycline antibiotic	tetracycline-resistant ribosomal protection protein
SPDS01000001.1_1198	1288888	1290078	+	*Escherichia coli* EF-Tu mutants conferring resistance to Pulvomycin	elfamycin antibiotic	elfamycin resistant EF-Tu
SPDS01000001.1_1238	1319060	1320430	+	ugd	peptide antibiotic	pmr phosphoethanolamine transferase
SPDS01000001.1_1244	1326210	1327409	+	vanTG	glycopeptide antibiotic	glycopeptide resistance gene cluster; vanT
SPDS01000001.1_1247	1328647	1329138	+	aacA43	aminoglycoside antibiotic	AAC(6')
SPDS01000001.1_1259	1341767	1342684	+	macB	macrolide antibiotic	ATP-binding cassette (ABC) antibiotic efflux pump
SPDS01000001.1_1260	1342681	1343628	+	RanA	aminoglycoside antibiotic	ATP-binding cassette (ABC) antibiotic efflux pump

SPDS01000001.1_1282	1372117	1372737	+	AAC(6')-Isa	aminoglycoside antibiotic	AAC(6')
SPDS01000001.1_1309	1390957	1391808	+	VIM-34	carbapenem; cephalosporin; cephamycin; penam; penem	VIM beta-lactamase
SPDS01000001.1_1345	1417585	1419492	+	otr(A)	tetracycline antibiotic	tetracycline-resistant ribosomal protection protein
SPDS01000001.1_1358	1429506	1430279	-	macB	macrolide antibiotic	ATP-binding cassette (ABC) antibiotic efflux pump
SPDS01000001.1_1376	1445138	1445761	-	MexL	macrolide antibiotic; tetracycline antibiotic; triclosan	resistance-nodulation-cell division (RND) antibiotic efflux pump
SPDS01000001.1_1388	1458035	1459027	-	RanA	aminoglycoside antibiotic	ATP-binding cassette (ABC) antibiotic efflux pump
SPDS01000001.1_1393	1466982	1468262	-	mphK	macrolide antibiotic	macrolide phosphotransferase (MPH)
SPDS01000001.1_1412	1490578	1491504	-	sul4	sulfonamide antibiotic	sulfonamide resistant sul
SPDS01000001.1_1425	1504383	1506074	+	macB	macrolide antibiotic	ATP-binding cassette (ABC) antibiotic efflux pump
SPDS01000001.1_1428	1507750	1508430	+	mtrA	macrolide antibiotic; penam	resistance-nodulation-cell division (RND) antibiotic efflux pump
SPDS01000001.1_1429	1508427	1510247	+	cpxA	aminoglycoside antibiotic; aminocoumarin antibiotic	resistance-nodulation-cell division (RND) antibiotic efflux pump
SPDS01000001.1_1450	1536450	1538228	+	*Streptococcus pneumoniae* PBP2x conferring resistance to amoxicillin	cephalosporin; cephamycin; penam	Penicillin-binding protein mutations conferring resistance to beta-lactam antibiotics
SPDS01000001.1_1512	1601703	1605026	+	efrB	macrolide antibiotic; fluoroquinolone antibiotic; rifamycin antibiotic	ATP-binding cassette (ABC) antibiotic efflux pump
SPDS01000001.1_1560	1655638	1656570	+	arnA	peptide antibiotic	pmr phosphoethanolamine transferase
SPDS01000001.1_1586	1684626	1685243	+	mtrA	macrolide antibiotic; penam	resistance-nodulation-cell division (RND) antibiotic efflux pump
SPDS01000001.1_1612	1712954	1713607	-	SPG-1	carbapenem	SPG beta-lactamase
SPDS01000001.1_1614	1714206	1717076	+	lmrC	macrolide antibiotic; lincosamide antibiotic; streptogramin antibiotic; tetracycline antibiotic; oxazolidinone antibiotic; phenicol antibiotic; pleuromutilin antibiotic	ABC-F ATP-binding cassette ribosomal protection protein
SPDS01000001.1_1640	1744143	1745030	-	tetA(58)	tetracycline antibiotic	major facilitator superfamily (MFS) antibiotic efflux pump
SPDS01000001.1_1644	1748874	1749632	+	novA	aminocoumarin antibiotic	ATP-binding cassette (ABC) antibiotic efflux pump
SPDS01000001.1_1650	1753984	1754673	-	patA	fluoroquinolone antibiotic	ATP-binding cassette (ABC) antibiotic efflux pump
SPDS01000001.1_1658	1761186	1762868	+	TaeA	pleuromutilin antibiotic	ATP-binding cassette (ABC) antibiotic efflux pump

SPDS01000001.1_1660	1763577	1764083	+	*Pseudomonas aeruginosa* soxR	fluoroquinolone antibiotic; cephalosporin; glycylcycline; penam; tetracycline antibiotic; acridine dye; rifamycin antibiotic; phenicol antibiotic; triclosan	ATP-binding cassette (ABC) antibiotic efflux pump; major facilitator superfamily (MFS) antibiotic efflux pump; resistance-nodulation-cell division (RND) antibiotic efflux pump
SPDS01000001.1_1675	1781939	1784557	-	*Staphylococcus aureus* mupB conferring resistance to mupirocin	mupirocin	antibiotic-resistant isoleucyl-tRNA synthetase (ileS)
SPDS01000001.1_1681	1789721	1791016	+	*Planobispora rosea* EF-Tu mutants conferring resistance to inhibitor GE2270A	elfamycin antibiotic	elfamycin resistant EF-Tu
SPDS01000001.1_1683	1792100	1792843	+	macB	macrolide antibiotic	ATP-binding cassette (ABC) antibiotic efflux pump
SPDS01000001.1_1690	1800071	1800688	-	evgA	macrolide antibiotic; fluoroquinolone antibiotic; penam; tetracycline antibiotic	major facilitator superfamily (MFS) antibiotic efflux pump; resistance-nodulation-cell division (RND) antibiotic efflux pump
SPDS01000001.1_1693	1802987	1806247	+	*Bifidobacterium bifidum* ileS conferring resistance to mupirocin	mupirocin	antibiotic-resistant isoleucyl-tRNA synthetase (ileS)
SPDS01000001.1_1699	1810626	1811210	-	AAC(6')-34	aminoglycoside antibiotic	AAC(6')
SPDS01000001.1_1740	1854340	1854966	+	QnrVC7	fluoroquinolone antibiotic	quinolone resistance protein (qnr)
SPDS01000001.1_1741	1854950	1855870	-	adeL	fluoroquinolone antibiotic; tetracycline antibiotic	resistance-nodulation-cell division (RND) antibiotic efflux pump
SPDS01000001.1_1744	1858390	1860072	-	srmB	macrolide antibiotic; lincosamide antibiotic; streptogramin antibiotic; tetracycline antibiotic; oxazolidinone antibiotic; phenicol antibiotic; pleuromutilin antibiotic	ABC-F ATP-binding cassette ribosomal protection protein
SPDS01000001.1_1767	1883262	1883891	-	BJP-1	carbapenem	BJP beta-lactamase
SPDS01000001.1_1773	1890955	1892433	-	tet(V)	tetracycline antibiotic	major facilitator superfamily (MFS) antibiotic efflux pump
SPDS01000001.1_1778	1898050	1899312	-	cmx	phenicol antibiotic	major facilitator superfamily (MFS) antibiotic efflux pump
SPDS01000001.1_1790	1911070	1911480	+	YajC	fluoroquinolone antibiotic; cephalosporin; glycylcycline; penam; tetracycline antibiotic; rifamycin antibiotic; phenicol antibiotic; triclosan	resistance-nodulation-cell division (RND) antibiotic efflux pump
SPDS01000001.1_1792	1913276	1914244	+	oqxB	fluoroquinolone antibiotic; glycylcycline; tetracycline antibiotic; diaminopyrimidine antibiotic; nitrofuran antibiotic	resistance-nodulation-cell division (RND) antibiotic efflux pump
SPDS01000001.1_1821	1942806	1946084	+	vanM	glycopeptide antibiotic	glycopeptide resistance gene cluster; van ligase

SPDS01000001.1_1835	1961043	1962224	+	*Bifidobacterium bifidum* ileS conferring resistance to mupirocin	mupirocin	antibiotic-resistant isoleucyl-tRNA synthetase (ileS)
SPDS01000001.1_1836	1962221	1963531	+	*Staphylococcus aureus* mupA conferring resistance to mupirocin	mupirocin	antibiotic-resistant isoleucyl-tRNA synthetase (ileS)
SPDS01000001.1_1844	1969700	1971547	+	tetB(P)	tetracycline antibiotic	tetracycline-resistant ribosomal protection protein
SPDS01000001.1_1859	1983095	1984480	-	tet(52)	tetracycline antibiotic	tetracycline inactivation enzyme
SPDS01000001.1_1887	2010962	2011813	+	chrB	macrolide antibiotic; lincosamide antibiotic	non-erm 23S ribosomal RNA methyltransferase (G748)
SPDS01000001.1_1890	2013575	2013922	+	RbpA	rifamycin antibiotic	RbpA bacterial RNA polymerase-binding protein
SPDS01000001.1_1891	2014137	2014865	-	PmrF	peptide antibiotic	pmr phosphoethanolamine transferase
SPDS01000001.1_1910	2035345	2036169	-	bacA	peptide antibiotic	undecaprenyl pyrophosphate related proteins
SPDS01000001.1_1916	2040292	2041728	-	cpxA	aminoglycoside antibiotic; aminocoumarin antibiotic	resistance-nodulation-cell division (RND) antibiotic efflux pump
SPDS01000001.1_1917	2041725	2042393	-	ParR	macrolide antibiotic; fluoroquinolone antibiotic; monobactam; aminoglycoside antibiotic; carbapenem; cephalosporin; cephamycin; penam; tetracycline antibiotic; acridine dye; phenicol antibiotic; penem	resistance-nodulation-cell division (RND) antibiotic efflux pump; Outer Membrane Porin (Opr)
SPDS01000001.1_1921	2047005	2048189	+	LpsB	peptide antibiotic	Intrinsic peptide antibiotic resistant Lps
SPDS01000001.1_1925	2051822	2052481	+	adeN	macrolide antibiotic; fluoroquinolone antibiotic; lincosamide antibiotic; carbapenem; cephalosporin; tetracycline antibiotic; rifamycin antibiotic; diaminopyrimidine antibiotic; phenicol antibiotic; penem	resistance-nodulation-cell division (RND) antibiotic efflux pump
SPDS01000001.1_1937	2062618	2063412	-	RanA	aminoglycoside antibiotic	ATP-binding cassette (ABC) antibiotic efflux pump
SPDS01000001.1_1938	2063409	2065430	-	macB	macrolide antibiotic	ATP-binding cassette (ABC) antibiotic efflux pump
SPDS01000001.1_1948	2074179	2074982	-	tsnR	peptide antibiotic	non-erm 23S ribosomal RNA methyltransferase (A1067)
SPDS01000001.1_1950	2075844	2076728	-	tetA(58)	tetracycline antibiotic	major facilitator superfamily (MFS) antibiotic efflux pump
SPDS01000001.1_1952	2077901	2079649	+	vanWI	glycopeptide antibiotic	vanW; glycopeptide resistance gene cluster
SPDS01000001.1_1960	2084403	2086004	-	poxtA	macrolide antibiotic; lincosamide antibiotic; streptogramin antibiotic; tetracycline antibiotic;	ABC-F ATP-binding cassette ribosomal protection protein

					oxazolidinone antibiotic; phenicol antibiotic; pleuromutilin antibiotic	
SPDS01000001.1_2040	2152653	2154914	+	macB	macrolide antibiotic	ATP-binding cassette (ABC) antibiotic efflux pump
SPDS01000001.1_2046	2157555	2158145	+	adeN	macrolide antibiotic; fluoroquinolone antibiotic; lincosamide antibiotic; carbapenem; cephalosporin; tetracycline antibiotic; rifamycin antibiotic; diaminopyrimidine antibiotic; phenicol antibiotic; penem	resistance-nodulation-cell division (RND) antibiotic efflux pump
SPDS01000001.1_2055	2165792	2167012	-	*Acinetobacter baumannii* AbaF	fosfomycin	major facilitator superfamily (MFS) antibiotic efflux pump
SPDS01000001.1_2058	2169061	2170281	+	rgt1438	rifamycin antibiotic	rifampin glycosyltransferase
SPDS01000001.1_2059	2170348	2171574	+	Erm(37)	macrolide antibiotic; lincosamide antibiotic; streptogramin antibiotic	Erm 23S ribosomal RNA methyltransferase
SPDS01000001.1_2100	2207050	2207823	+	catB3	phenicol antibiotic	chloramphenicol acetyltransferase (CAT)
SPDS01000001.1_2122	2229362	2231071	+	otr(B)	tetracycline antibiotic	major facilitator superfamily (MFS) antibiotic efflux pump
SPDS01000001.1_2133	2245924	2247159	+	emeA	acridine dye	multidrug and toxic compound extrusion (MATE) transporter
SPDS01000001.1_2153	2273652	2274539	-	tetA(58)	tetracycline antibiotic	major facilitator superfamily (MFS) antibiotic efflux pump
SPDS01000001.1_2165	2285695	2286807	-	D-Ala-D-Ala ligase	glycopeptide antibiotic	van ligase
SPDS01000001.1_2168	2288654	2289355	-	*Chlamydia trachomatis* intrinsic murA conferring resistance to fosfomycin	fosfomycin	antibiotic-resistant murA transferase
SPDS01000001.1_2169	2289502	2289972	-	*Chlamydia trachomatis* intrinsic murA conferring resistance to fosfomycin	fosfomycin	antibiotic-resistant murA transferase
SPDS01000001.1_2182	2307098	2307790	+	adeR	glycylcycline; tetracycline antibiotic	resistance-nodulation-cell division (RND) antibiotic efflux pump
SPDS01000001.1_2183	2307860	2309410	-	*Staphylococcus aureus* mupA conferring resistance to mupirocin	mupirocin	antibiotic-resistant isoleucyl-tRNA synthetase (ileS)
SPDS01000001.1_2198	2332318	2333910	-	vanHD	glycopeptide antibiotic	vanH; glycopeptide resistance gene cluster
SPDS01000001.1_2200	2334344	2335429	+	LpsB	peptide antibiotic	Intrinsic peptide antibiotic resistant Lps
SPDS01000001.1_2233	2381033	2381566	-	emrR	fluoroquinolone antibiotic	major facilitator superfamily (MFS) antibiotic efflux pump
SPDS01000001.1_2238	2386191	2387123	+	vanHB	glycopeptide antibiotic	vanH; glycopeptide resistance gene cluster

SPDS01000001.1_2243	2391959	2395336	+	MuxB	macrolide antibiotic; monobactam; tetracycline antibiotic; aminocoumarin antibiotic	resistance-nodulation-cell division (RND) antibiotic efflux pump
SPDS01000001.1_2245	2396302	2397048	+	adeR	glycylcycline; tetracycline antibiotic	resistance-nodulation-cell division (RND) antibiotic efflux pump
SPDS01000001.1_2251	2400735	2401250	-	AAC(6')-Ia	aminoglycoside antibiotic	AAC(6')
SPDS01000001.1_2252	2401689	2403116	+	vanXYC	glycopeptide antibiotic	glycopeptide resistance gene cluster; vanXY
SPDS01000001.1_2266	2418413	2419120	+	macB	macrolide antibiotic	ATP-binding cassette (ABC) antibiotic efflux pump
SPDS01000001.1_2267	2419117	2420352	+	macB	macrolide antibiotic	ATP-binding cassette (ABC) antibiotic efflux pump
SPDS01000001.1_2276	2429482	2431122	+	tlrC	macrolide antibiotic; lincosamide antibiotic; streptogramin antibiotic; tetracycline antibiotic; oxazolidinone antibiotic; phenicol antibiotic; pleuromutilin antibiotic	ABC-F ATP-binding cassette ribosomal protection protein
SPDS01000001.1_2279	2432833	2434389	-	facT	elfamycin antibiotic	major facilitator superfamily (MFS) antibiotic efflux pump
SPDS01000001.1_2287	2443507	2444847	-	*Acinetobacter baumannii* AbaF	fosfomycin	major facilitator superfamily (MFS) antibiotic efflux pump
SPDS01000001.1_2296	2455166	2456083	-	tetA(58)	tetracycline antibiotic	major facilitator superfamily (MFS) antibiotic efflux pump
SPDS01000001.1_2311	2469122	2469880	-	tetA(58)	tetracycline antibiotic	major facilitator superfamily (MFS) antibiotic efflux pump
SPDS01000001.1_2339	2498601	2500244	-	*Staphylococcus aureus* mupA conferring resistance to mupirocin	mupirocin	antibiotic-resistant isoleucyl-tRNA synthetase (ileS)
SPDS01000001.1_2342	2502514	2503305	-	macB	macrolide antibiotic	ATP-binding cassette (ABC) antibiotic efflux pump
SPDS01000001.1_2354	2515858	2516691	-	bcrA	peptide antibiotic	ATP-binding cassette (ABC) antibiotic efflux pump
SPDS01000001.1_2366	2530106	2530567	+	CrcB	aminoglycoside antibiotic	multidrug and toxic compound extrusion (MATE) transporter
SPDS01000001.1_2373	2535089	2535886	-	macB	macrolide antibiotic	ATP-binding cassette (ABC) antibiotic efflux pump
SPDS01000001.1_2392	2555640	2559236	-	vanO	glycopeptide antibiotic	glycopeptide resistance gene cluster; van ligase
SPDS01000001.1_2396	2561717	2562472	-	golS	monobactam; carbapenem; cephalosporin; cephamycin; penam; phenicol antibiotic; penem	resistance-nodulation-cell division (RND) antibiotic efflux pump
SPDS01000001.1_2399	2563869	2565758	-	iri	rifamycin antibiotic	rifampin monooxygenase
SPDS01000001.1_2400	2566001	2567707	-	srmB	macrolide antibiotic; lincosamide antibiotic; streptogramin antibiotic; tetracycline antibiotic;	ABC-F ATP-binding cassette ribosomal protection protein

					oxazolidinone antibiotic; phenicol antibiotic; pleuromutilin antibiotic	
SPDS01000001.1_2414	2579782	2580633	+	adeS	glycylcycline; tetracycline antibiotic	resistance-nodulation-cell division (RND) antibiotic efflux pump
SPDS01000001.1_2415	2580649	2581398	+	adeR	glycylcycline; tetracycline antibiotic	resistance-nodulation-cell division (RND) antibiotic efflux pump
SPDS01000001.1_2435	2606387	2607334	-	tsnR	peptide antibiotic	non-erm 23S ribosomal RNA methyltransferase (A1067)
SPDS01000002.1_5	9416	10483	-	RanA	aminoglycoside antibiotic	ATP-binding cassette (ABC) antibiotic efflux pump
SPDS01000002.1_22	27475	28884	-	baeS	aminoglycoside antibiotic; aminocoumarin antibiotic	resistance-nodulation-cell division (RND) antibiotic efflux pump
SPDS01000002.1_23	29066	29773	-	mtrA	macrolide antibiotic; penam	resistance-nodulation-cell division (RND) antibiotic efflux pump
SPDS01000002.1_39	45079	46611	-	*Acinetobacter baumannii* AbaF	fosfomycin	major facilitator superfamily (MFS) antibiotic efflux pump
SPDS01000002.1_52	60055	61614	+	otr(B)	tetracycline antibiotic	major facilitator superfamily (MFS) antibiotic efflux pump
SPDS01000002.1_66	73625	74374	+	macB	macrolide antibiotic	ATP-binding cassette (ABC) antibiotic efflux pump
SPDS01000002.1_90	100276	100845	+	arnA	peptide antibiotic	pmr phosphoethanolamine transferase
SPDS01000002.1_95	106566	107135	+	AcrS	fluoroquinolone antibiotic; cephalosporin; glycylcycline; cephamycin; penam; tetracycline antibiotic; rifamycin antibiotic; phenicol antibiotic; triclosan	resistance-nodulation-cell division (RND) antibiotic efflux pump
SPDS01000002.1_96	107140	108477	-	*Rhodococcus fascians* cmr	phenicol antibiotic	major facilitator superfamily (MFS) antibiotic efflux pump
SPDS01000002.1_101	113745	115205	-	lmrB	lincosamide antibiotic	ATP-binding cassette (ABC) antibiotic efflux pump
SPDS01000002.1_110	123313	124179	-	vanHA	glycopeptide antibiotic	vanH; glycopeptide resistance gene cluster
SPDS01000002.1_138	170854	171723	+	marA	fluoroquinolone antibiotic; monobactam; carbapenem; cephalosporin; glycylcycline; cephamycin; penam; tetracycline antibiotic; rifamycin antibiotic; phenicol antibiotic; triclosan; penem	resistance-nodulation-cell division (RND) antibiotic efflux pump; General Bacterial Porin with reduced permeability to beta-lactams
SPDS01000002.1_140	173561	174079	-	apmA	aminoglycoside antibiotic	amp acetyltransferase
SPDS01000002.1_144	177222	178607	-	*Acinetobacter baumannii* AbaF	fosfomycin	major facilitator superfamily (MFS) antibiotic efflux pump
SPDS01000002.1_145	178735	179634	+	adeL	fluoroquinolone antibiotic; tetracycline antibiotic	resistance-nodulation-cell division (RND) antibiotic efflux pump

SPDS01000002.1_147	179971	180633	-	evgA	macrolide antibiotic; fluoroquinolone antibiotic; penam; tetracycline antibiotic	major facilitator superfamily (MFS) antibiotic efflux pump; resistance-nodulation-cell division (RND) antibiotic efflux pump
SPDS01000002.1_162	195089	196564	+	macB	macrolide antibiotic	ATP-binding cassette (ABC) antibiotic efflux pump
SPDS01000002.1_218	263409	264293	+	Erm(38)	macrolide antibiotic; lincosamide antibiotic; streptogramin antibiotic	Erm 23S ribosomal RNA methyltransferase
SPDS01000002.1_223	267856	269694	-	TaeA	pleuromutilin antibiotic	ATP-binding cassette (ABC) antibiotic efflux pump
SPDS01000002.1_247	294908	296563	+	oleB	macrolide antibiotic; lincosamide antibiotic; streptogramin antibiotic; tetracycline antibiotic; oxazolidinone antibiotic; phenicol antibiotic; pleuromutilin antibiotic	ABC-F ATP-binding cassette ribosomal protection protein
SPDS01000002.1_248	296560	297825	+	tet(V)	tetracycline antibiotic	major facilitator superfamily (MFS) antibiotic efflux pump
SPDS01000002.1_249	298336	299724	+	*Acinetobacter baumannii* AbaF	fosfomycin	major facilitator superfamily (MFS) antibiotic efflux pump
SPDS01000002.1_251	300820	302601	-	vanE	glycopeptide antibiotic	glycopeptide resistance gene cluster; van ligase
SPDS01000002.1_285	336503	337102	-	AAC(6')-Isa	aminoglycoside antibiotic	AAC(6')
SPDS01000002.1_305	349408	350586	+	cmlv	phenicol antibiotic	chloramphenicol phosphotransferase
SPDS01000002.1_319	365347	366207	-	vph	peptide antibiotic	viomycin phosphotransferase
SPDS01000002.1_321	367090	367527	+	AAC(6')-Iak	aminoglycoside antibiotic	AAC(6')
SPDS01000002.1_337	381117	381917	-	macB	macrolide antibiotic	ATP-binding cassette (ABC) antibiotic efflux pump
SPDS01000002.1_347	393550	395070	+	vanSO	glycopeptide antibiotic	vanS; glycopeptide resistance gene cluster
SPDS01000002.1_379	428550	429053	-	*Escherichia coli* marR mutant conferring antibiotic resistance	fluoroquinolone antibiotic; cephalosporin; glycylcycline; penam; tetracycline antibiotic; rifamycin antibiotic; phenicol antibiotic; triclosan	resistance-nodulation-cell division (RND) antibiotic efflux pump
SPDS01000002.1_383	431933	432649	-	ParR	macrolide antibiotic; fluoroquinolone antibiotic; monobactam; aminoglycoside antibiotic; carbapenem; cephalosporin; cephamycin; penam; tetracycline antibiotic; acridine dye; phenicol antibiotic; penem	resistance-nodulation-cell division (RND) antibiotic efflux pump; Outer Membrane Porin (Opr)
SPDS01000002.1_384	432679	433959	-	basS	peptide antibiotic	pmr phosphoethanolamine transferase
SPDS01000002.1_394	444170	445813	+	RanA	aminoglycoside antibiotic	ATP-binding cassette (ABC) antibiotic efflux pump
SPDS01000002.1_399	450830	452527	+	RanA	aminoglycoside antibiotic	ATP-binding cassette (ABC) antibiotic efflux pump

SPDS01000002.1_406	457677	458297	-	adeN	macrolide antibiotic; fluoroquinolone antibiotic; lincosamide antibiotic; carbapenem; cephalosporin; tetracycline antibiotic; rifamycin antibiotic; diaminopyrimidine antibiotic; phenicol antibiotic; penem	resistance-nodulation-cell division (RND) antibiotic efflux pump
SPDS01000002.1_414	465224	466036	+	AAC(6')-Ih	aminoglycoside antibiotic	AAC(6')
SPDS01000002.1_429	480803	482650	+	otr(A)	tetracycline antibiotic	tetracycline-resistant ribosomal protection protein
SPDS01000002.1_444	494150	494926	-	macB	macrolide antibiotic	ATP-binding cassette (ABC) antibiotic efflux pump
SPDS01000002.1_457	507033	508643	+	basS	peptide antibiotic	pmr phosphoethanolamine transferase
SPDS01000002.1_458	508654	509328	+	*Pseudomonas aeruginosa* CpxR	macrolide antibiotic; fluoroquinolone antibiotic; monobactam; aminoglycoside antibiotic; carbapenem; cephalosporin; cephamycin; penam; tetracycline antibiotic; peptide antibiotic; aminocoumarin antibiotic; diaminopyrimidine antibiotic; sulfonamide antibiotic; phenicol antibiotic; penem	resistance-nodulation-cell division (RND) antibiotic efflux pump
SPDS01000002.1_488	544985	545812	+	macB	macrolide antibiotic	ATP-binding cassette (ABC) antibiotic efflux pump
SPDS01000002.1_498	554806	555591	+	tlrC	macrolide antibiotic; lincosamide antibiotic; streptogramin antibiotic; tetracycline antibiotic; oxazolidinone antibiotic; phenicol antibiotic; pleuromutilin antibiotic	ABC-F ATP-binding cassette ribosomal protection protein
SPDS01000002.1_530	585645	586499	+	tsnR	peptide antibiotic	non-erm 23S ribosomal RNA methyltransferase (A1067)
SPDS01000003.1_3	5432	6379	-	tsnR	peptide antibiotic	non-erm 23S ribosomal RNA methyltransferase (A1067)
SPDS01000003.1_8	10780	11460	-	mtrA	macrolide antibiotic; penam	resistance-nodulation-cell division (RND) antibiotic efflux pump
SPDS01000003.1_9	11457	12662	-	cpxA	aminoglycoside antibiotic; aminocoumarin antibiotic	resistance-nodulation-cell division (RND) antibiotic efflux pump
SPDS01000003.1_45	54948	55559	+	*Escherichia coli* acrR with mutation conferring multidrug antibiotic resistance	fluoroquinolone antibiotic; cephalosporin; glycylcycline; penam; tetracycline antibiotic; rifamycin antibiotic; phenicol antibiotic; triclosan	resistance-nodulation-cell division (RND) antibiotic efflux pump
SPDS01000003.1_46	55556	57169	-	efrA	macrolide antibiotic; fluoroquinolone antibiotic; rifamycin antibiotic	ATP-binding cassette (ABC) antibiotic efflux pump
SPDS01000003.1_49	59129	59683	-	vanRO	glycopeptide antibiotic	glycopeptide resistance gene cluster; vanR
SPDS01000003.1_50	60529	61269	+	mtrA	macrolide antibiotic; penam	resistance-nodulation-cell division (RND) antibiotic efflux pump

SPDS01000003.1_51	61266	62777	+	vanSM	glycopeptide antibiotic	vanS; glycopeptide resistance gene cluster
SPDS01000003.1_63	71018	71770	-	msbA	nitroimidazole antibiotic	ATP-binding cassette (ABC) antibiotic efflux pump
SPDS01000003.1_105	106936	108129	+	tet(43)	tetracycline antibiotic	major facilitator superfamily (MFS) antibiotic efflux pump
SPDS01000003.1_109	112481	113677	+	iri	rifamycin antibiotic	rifampin monooxygenase
SPDS01000003.1_122	124868	125839	+	vanHM	glycopeptide antibiotic	vanH; glycopeptide resistance gene cluster
SPDS01000003.1_134	137725	139173	-	lmrB	lincosamide antibiotic	ATP-binding cassette (ABC) antibiotic efflux pump
SPDS01000003.1_140	144576	145271	-	YojI	peptide antibiotic	ATP-binding cassette (ABC) antibiotic efflux pump
SPDS01000003.1_142	146408	147115	+	adeN	macrolide antibiotic; fluoroquinolone antibiotic; lincosamide antibiotic; carbapenem; cephalosporin; tetracycline antibiotic; rifamycin antibiotic; diaminopyrimidine antibiotic; phenicol antibiotic; penem	resistance-nodulation-cell division (RND) antibiotic efflux pump
SPDS01000003.1_157	164379	165053	+	evgA	macrolide antibiotic; fluoroquinolone antibiotic; penam; tetracycline antibiotic	major facilitator superfamily (MFS) antibiotic efflux pump; resistance-nodulation-cell division (RND) antibiotic efflux pump
SPDS01000003.1_181	193009	194631	+	tcr3	tetracycline antibiotic	major facilitator superfamily (MFS) antibiotic efflux pump
SPDS01000003.1_187	199572	200177	+	APH(3')-IIc	aminoglycoside antibiotic	APH(3')
SPDS01000003.1_188	200190	200861	-	mtrA	macrolide antibiotic; penam	resistance-nodulation-cell division (RND) antibiotic efflux pump
SPDS01000003.1_189	200858	202564	-	ParS	macrolide antibiotic; fluoroquinolone antibiotic; monobactam; aminoglycoside antibiotic; carbapenem; cephalosporin; cephamycin; penam; tetracycline antibiotic; acridine dye; phenicol antibiotic; penem	resistance-nodulation-cell division (RND) antibiotic efflux pump; Outer Membrane Porin (Opr)
SPDS01000003.1_217	232011	233633	-	macB	macrolide antibiotic	ATP-binding cassette (ABC) antibiotic efflux pump
SPDS01000003.1_224	239289	240308	-	RanA	aminoglycoside antibiotic	ATP-binding cassette (ABC) antibiotic efflux pump
SPDS01000003.1_229	246106	246924	-	tetA(60)	tetracycline antibiotic	ATP-binding cassette (ABC) antibiotic efflux pump
SPDS01000003.1_237	256579	257400	+	macB	macrolide antibiotic	ATP-binding cassette (ABC) antibiotic efflux pump
SPDS01000003.1_256	276937	278241	+	tet(V)	tetracycline antibiotic	major facilitator superfamily (MFS) antibiotic efflux pump

SPDS01000003.1_267	287685	288482	+	golS	monobactam; carbapenem; cephalosporin; cephamycin; penam; phenicol antibiotic; penem	resistance-nodulation-cell division (RND) antibiotic efflux pump
SPDS01000003.1_278	299035	300429	+	arlS	fluoroquinolone antibiotic; acridine dye	major facilitator superfamily (MFS) antibiotic efflux pump
SPDS01000003.1_279	300426	301094	+	arlR	fluoroquinolone antibiotic; acridine dye	major facilitator superfamily (MFS) antibiotic efflux pump
SPDS01000003.1_285	306047	306703	-	tsnR	peptide antibiotic	non-erm 23S ribosomal RNA methyltransferase (A1067)
SPDS01000003.1_287	307848	308402	+	MexL	macrolide antibiotic; tetracycline antibiotic; triclosan	resistance-nodulation-cell division (RND) antibiotic efflux pump
SPDS01000003.1_288	308495	309220	+	tetA(58)	tetracycline antibiotic	major facilitator superfamily (MFS) antibiotic efflux pump
SPDS01000001.1_149	154609	157203	-	*Clostridioides difficile* gyrA conferring resistance to fluoroquinolones	fluoroquinolone antibiotic	fluoroquinolone resistant gyrA
SPDS01000001.1_150	157295	159358	-	*Clostridioides difficile* gyrB conferring resistance to fluoroquinolones	fluoroquinolone antibiotic	fluoroquinolone resistant gyrB
SPDS01000001.1_348	368801	370783	-	*Mycobacterium tuberculosis* ethA mutations conferring resistance to isoniazid	isoniazid	isoniazid resistant ethA
SPDS01000001.1_360	381070	383052	+	*Mycobacterium tuberculosis* ethA mutations conferring resistance to isoniazid	isoniazid	isoniazid resistant ethA
SPDS01000001.1_1020	1094202	1095698	+	*Enterococcus faecalis* cls with mutation conferring resistance to daptomycin	peptide antibiotic	daptomycin resistant cls
SPDS01000001.1_1507	1595101	1597209	+	*Mycobacterium tuberculosis* gyrB mutant conferring resistance to fluoroquinolones	fluoroquinolone antibiotic	fluoroquinolone resistant gyrB
SPDS01000001.1_1513	1605096	1607729	-	*Escherichia coli* gyrA conferring resistance to fluoroquinolones	fluoroquinolone antibiotic	fluoroquinolone resistant gyrA
SPDS01000001.1_1694	1806247	1807599	+	*Mycobacterium tuberculosis* folC with mutation conferring resistance to para-aminosalicylic acid	para-aminosalicylic acid	aminosalicylate resistant dihydrofolate synthase
SPDS01000001.1_2071	2181861	2183111	+	*Mycobacterium tuberculosis* kasA mutant conferring resistance to isoniazid	isoniazid	antibiotic resistant kasA

SPDS01000001.1_2307	2464355	2464948	+	*Mycobacterium tuberculosis* pncA mutations conferring resistance to pyrazinamide	pyrazinamide	pyrazinamide resistant pncA
SPDS01000002.1_295	343845	344417	+	*Mycobacterium tuberculosis* pncA mutations conferring resistance to pyrazinamide	pyrazinamide	pyrazinamide resistant pncA
SPDS01000003.1_244	264501	265154	+	*Mycobacterium tuberculosis* katG mutations conferring resistance to isoniazid	isoniazid	isoniazid resistant katG
SPDS01000003.1_245	265145	266680	+	*Mycobacterium tuberculosis* katG mutations conferring resistance to isoniazid	isoniazid	isoniazid resistant katG
SPDS01000001.1_232	246690	247169	+	MexZ	macrolide antibiotic; fluoroquinolone antibiotic; aminoglycoside antibiotic; carbapenem; cephalosporin; cephamycin; penam; tetracycline antibiotic; acridine dye; phenicol antibiotic	resistance-nodulation-cell division (RND) antibiotic efflux pump
SPDS01000001.1_361	383264	383800	+	nalD	macrolide antibiotic; fluoroquinolone antibiotic; monobactam; carbapenem; cephalosporin; cephamycin; penam; tetracycline antibiotic; peptide antibiotic; aminocoumarin antibiotic; diaminopyrimidine antibiotic; sulfonamide antibiotic; phenicol antibiotic; penem	resistance-nodulation-cell division (RND) antibiotic efflux pump
SPDS01000001.1_576	620669	621025	+	*Escherichia coli* marR mutant conferring antibiotic resistance	fluoroquinolone antibiotic; cephalosporin; glycylcycline; penam; tetracycline antibiotic; rifamycin antibiotic; phenicol antibiotic; triclosan	resistance-nodulation-cell division (RND) antibiotic efflux pump
SPDS01000001.1_610	652954	653928	+	MexS	fluoroquinolone antibiotic; diaminopyrimidine antibiotic; phenicol antibiotic	resistance-nodulation-cell division (RND) antibiotic efflux pump
SPDS01000001.1_782	837565	838212	-	tetR	tetracycline antibiotic	major facilitator superfamily (MFS) antibiotic efflux pump
SPDS01000001.1_878	936132	937106	-	MexT	fluoroquinolone antibiotic; diaminopyrimidine antibiotic; phenicol antibiotic	resistance-nodulation-cell division (RND) antibiotic efflux pump
SPDS01000001.1_2247	2398204	2398662	+	MexR	macrolide antibiotic; fluoroquinolone antibiotic; monobactam; carbapenem; cephalosporin; cephamycin; penam; tetracycline antibiotic; peptide antibiotic; aminocoumarin antibiotic; diaminopyrimidine antibiotic; sulfonamide antibiotic; phenicol antibiotic; penem	resistance-nodulation-cell division (RND) antibiotic efflux pump
SPDS01000002.1_546	603708	604676	-	MexS	fluoroquinolone antibiotic; diaminopyrimidine antibiotic; phenicol antibiotic	resistance-nodulation-cell division (RND) antibiotic efflux pump
SPDS01000003.1_23	25534	26523	-	MexS	fluoroquinolone antibiotic; diaminopyrimidine antibiotic; phenicol antibiotic	resistance-nodulation-cell division (RND) antibiotic efflux pump

SPDS01000003.1_182	194640	195116	+	MexR	macrolide antibiotic; fluoroquinolone antibiotic; monobactam; carbapenem; cephalosporin; cephamycin; penam; tetracycline antibiotic; peptide antibiotic; aminocoumarin antibiotic; diaminopyrimidine antibiotic; sulfonamide antibiotic; phenicol antibiotic; penem	resistance-nodulation-cell division (RND) antibiotic efflux pump
SPDS01000003.1_272	293813	294346	+	tetR	tetracycline antibiotic	major facilitator superfamily (MFS) antibiotic efflux pump

Supplementary Table S6. Comparative list of genes linked to stress adaptation, hydrolytic activity, and PGP activity in the accessible genomes of the genus *Glutamicibacter* and its closely related genus *Arthrobacter*.

Strain name	Isolation source/type of stress	Gene category			Reference
		Stress adaptation	Hydrolytic enzymes	PGP attributes	
G. arilaitensis LJH19	Night soil compost/ cold stress	Cold-shock protein, Co-chaperone GroES, Chaperonin GroEL, Molecular chaperone DnaJ, Cold-shock protein, Co-chaperone GroES, Molecular chaperone DnaK, ATP-dependent chaperone ClpB, Heat shock protein HslJ / META domain-containing protein, Molecular chaperone DnaK, ATP-dependent chaperone ClpB, Carbon starvation protein A, 1-acyl-sn-glycerol-3-phosphate acyltransferase, 3-oxoacyl-ACP synthase III, Phytoene desaturase, Phytoene/squalene synthase, Universal stress protein, GlsB/YeaQ/YmgE family stress response membrane protein, Serine/threonine protein kinase, Glycine betaine ABC transporter substrate-binding protein, Sarcosine oxidase subunit beta family protein, Sarcosine oxidase subunit delta family protein, Sarcosine oxidase subunit alpha family protein, Sarcosine oxidase subunit gamma family protein, Superoxide dismutase, Catalase, OsmC family peroxiredoxin,	ATP-dependent Clp protease proteolytic subunit, ATP-dependent Clp protease ATP-binding subunit ClpX, Putative esterase, pullulanase-type alpha-1,6-glucosidase, trypsin-like serine protease,<br><br>MarP family serine protease, Alpha-amylase, alpha/beta fold hydrolase,<br><br>Beta-glucosidase, Xylose isomerase, spermidine/putrescine ABC transporter substrate-binding protein, amino acid permease, amino acid ABC transporter ATP-binding protein, phosphate ABC transporter ATP-binding protein, phosphate ABC transporter permease PstA, phosphate ABC transporter permease subunit PstC, phosphate ABC transporter substrate-binding protein PstS, phosphate/phosphite/phosphonate ABC transporter substrate-binding protein, phosphonate ABC transporter, permease protein PhnE, phosphonate ABC transporter ATP-binding protein, peptide ABC transporter substrate-binding protein	Amidase, Anthranilate synthase component I, Nitrite reductase [NAD(P)H], nitrite reductase (NAD(P)H) small subunit, nitrite reductase, nitrite/sulfite reductase, nitrate reductase, Isochorismate synthase, chorismate synthase, Anthranilate phosphoribosyl transferase, Isochorismatase family protein YecD, Acetylornithine aminotransferase, alkaline phosphatase, Inositol-1-monophosphatase, tryptophan synthase subunit beta, tryptophan synthase subunit alpha, indole-3-glycerol phosphate synthase TrpC, Ornithine carbamoyltransferase, ornithine decarboxylase, Argininosuccinate lyase, phosphoribosyl anthranilate isomerase PriA, Formimidoylglutamase* (Arginase), agmatinase, inorganic diphosphatase, short-chain fatty acid transporter, D-serine/D-alanine/glycine transporter, MFS transporter (nitrate), gluconate permease, iron-enterobactin ABC	This study

		Organic hydroperoxide resistance protein, Thioredoxin, Thioredoxin-disulfide reductase, Thioredoxin-dependent thiol peroxidase, Thioredoxin family protein, Thioredoxin domain-containing protein, Sodium/proline symporter PutP, Na+/H+ antiporter NhaA, Trehalose-6-phosphate synthase, Trehalose-phosphatase, Recombinase family protein, Recombinase RecA, Tyrosine recombinase XerC, Peroxide stress protein YaaA, 50S ribosomal protein L25, SOS response-associated peptidase, Type II toxin-antitoxin system prevent-host-death family antitoxin, Type II toxin-antitoxin system VapB family antitoxin, Type II toxin-antitoxin system HipA family toxin, Type II toxin-antitoxin system Phd/YefM family antitoxin, Type II toxin-antitoxin system PemK/MazF family toxin, Toxin component of a toxin/antitoxin system, Serine/threonine-protein kinase, Site-specific tyrosine recombinase XerD, Recombinase, ATP-dependent DNA helicase RecQ, DNA repair protein RadA, DNA repair protein RecO, DNA integrity scanning protein DisA, DNA repair protein RecN, ATP-dependent DNA helicase RecG, ATP-dependent DNA helicase UvrD2, Holliday junction branch migration protein RuvA, Holliday junction		transporter permease, Fe(3+)-siderophore ABC transporter permease, siderophore-interacting protein, Fe2+-enterobactin ABC transporter substrate-binding protein, glutamate synthase subunit beta, glutamate synthase large subunit, FMN-binding glutamate synthase family protein, Glutamine synthetase	

		branch migration DNA helicase RuvB, Holliday junction resolvase RuvX			
G. arilaitensis Re117T	Cheese surface/osmotic stress	Putative A/G-specific DNA glycosylase, DNA integrity scanning protein DisA, DNA repair protein RadA, pyrimidine dimer DNA glycosylase, exodeoxyribonuclease III<br><br>methylated-DNA--[protein]-cysteine S-methyltransferase, recombination protein RecR, putative DNA polymerase subunit UmuD, putative DNA polymerase subunit UmuC, DNA glycosylase, putative ATP-dependent DNA helicase, uracil-DNA glycosylase, putative DNA-3-methyladenine, glycosylase II, putative DNA-3-methyladenine glycosylase I, exodeoxyribonuclease III, transcription-repair-coupling factor, RecA bacterial DNA recombination protein<br><br>putative regulatory protein RecX, DNA repair protein RecN, putative ATP-dependent DNA helicase RecG, formamidopyrimidine-DNA glycosylase, putative 3-methyladenine DNA glycosylase, excinuclease ABC subunit A, DNA repair protein RecO, Holliday junction ATP-dependent DNA helicase RuvB, Holliday junction ATP-dependent DNA helicase RuvA, crossover junction endodeoxyribonuclease RuvC,	Rhomboid family protein, pyroglutamyl-peptidase I, putative cell division protein FtsH, putative metalloproteinase, putative membrane-bound M23 family peptidase, secreted subtilase family protease, putative secreted M23 family peptidase, prolyl oligopeptidase family protein, putative secreted peptidase, putative M23 family peptidase, putative metal-dependent amidase/aminoacylase/carboxypeptidase, membrane-associated subtilase family protease, putative transglutaminase-like protease, putative secreted M23 family peptidase, zinc metallopeptidase, putative M16 family peptidase, M1 family aminopeptidase, putative secreted M23 family peptidase, ATP-dependent Clp, protease adaptor protein ClpS, embrane alanyl aminopeptidase, putative prolyl aminopeptidase, putative zinc metallopeptidase, zinc metallopeptidase, putative ATP-dependent 26S proteasome regulatory subunit, putative proteasome component, putative proteasome component, putative zinc metallopeptidase, putative metalloprotease, peptidyl-dipeptidase, ATP-dependent Clp protease ATP-binding subunit ClpX, ATP-dependent Clp, protease proteolytic subunit ClpP, ATP-dependent Clp, protease proteolytic subunit ClpP, membrane alanyl aminopeptidase, putative prolyl aminopeptidase, leucyl aminopeptidase, putative serine protease,	ABC-type Fe3+/siderophore transport system, ATPase component; ABC-type Fe3+/siderophore transport system,<br><br>permease component; ABC-type Fe3+/siderophore transport system, substrate binding component; ABC-type Fe3+/siderophore<br><br>transport system, fused<br><br>permease and ATPase<br><br>component	(Monnet et al., 2010)

		DNA glycosylase, excinuclease ABC subunit C, excinuclease ABC subunit A, putative DNA polymerase IV, putative methylated-DNA--[protein]-cysteine S-methyltransferase, exodeoxyribonuclease VII large subunit, exodeoxyribonuclease VII small subunit, DNA-(apurinic or apyrimidinic site) lyase, excinuclease ABC subunit A, deoxyribodipyrimidine photo-lyase, putative very short patch repair endonuclease, UmuD-like protein, UmuC-like protein	Xaa-Pro aminopeptidase, probable O-sialoglycoprotein endopeptidase, putative glycoprotease, M18 family aminopeptidase, protease HtpX, homolog<br><br>putative oligopeptidase B, putative membrane-associated serine protease, prolyl oligopeptidase family protein, putative metal-dependent amidase/aminoacylase/carboxypeptidase, secreted subtilase family protease, prolyl oligopeptidase family protein, putative M23 family peptidase, PfpI family intracellular protease, putative carboxypeptidase, putative secreted M23 family peptidase, putative glycerophosphodiester phosphodiesterase, putative glycerophosphodiester phosphodiesterase, secreted triacylglycerol lipase<br><br>phospholipase/carboxylesterase family protein, putative glycerophosphodiester phosphodiesterase, GDSL-like esterase/lipase phospholipase/carboxylesterase family protein, GDSL-like esterase/lipase, putative esterase/lipase, putative secreted M23 family peptidase, putative esterase/lipase, putative esterase/lipase, putative esterase/lipase, putative esterase/lipase, GDSL-like esterase/lipase, possible lysophospholipase, GDSL-like esterase/lipase, putative periplasmic glycerophosphoryl diester phosphodiesterase		

G.halophytocola KLBMP 5180	Coastal halophyte/ salinity stress	Na+/H+ antiporter, K+/H+ antiporter subunit A/B, Na+/H+ antiporter subunit C, Na+/H+ antiporter subunit D, Cation/proton antiporter, Cation/proton antiporter, Sodium: proton antiporter ATP-binding protein Mrp, K+ efflux system protein, K+ transporter, Choline dehydrogenase, Betaine-aldehyde dehydrogenase, Glycine betaine transporter, Glycine betaine transporter, Glycine betaine/L-proline ABC transporter, Arsenic resistance protein, Copper resistance protein, Copper resistance protein C, Cobalt-zinc-cadmium resistance protein, Tellurium resistance protein,<br><br>UTP-glucose-1-phosphate uridylyltransferase, Galactose-1-phosphate uridylyltransferase (EC 2.7.7.12), UDP-galactopyranose mutase, UDP-N-acetylglucosamine transferase, UDP-glucose 4-epimerase, Mannose-1-phosphate guanylyltransferase, Undecaprenyl pyrophosphate synthase, Undecaprenyl-diphosphatase, Mannosyltransferase, Glycosyltransferase, Glycosyltransferase family 1, Glycosyltransferase family 2, Glycosyltransferase family 9, Carbohydrate esterase, carboxylesterase, Carbohydrate esterase, metal-dependent phosphoesterase, Carbohydrate	Phenylacetic acid degradation protein, 4-hydroxyphenylacetate degradation bifunctional isomerase/ Decarboxylase, Phenol 2-monooxygenase, 4-hydroxybenzoate 3-monooxygenase, Chitin deacetylase	Nitrogen fixation protein, Tryptophan 2-monooxygenase, Siderophore biosynthesis protein, Probable siderophore biosynthesis protein SbnA, Siderophore synthetase, Siderophore ABC transport system ATP-binding protein, Siderophore ABC transporter substrate-binding protein, Phenazine biosynthesis protein, Phenazine biosynthesis protein, Phenazine biosynthesis protein, GABA permease, 4-aminobutyrate aminotransferase, Agmatinase, Spermidine synthase, Cobalamin biosynthesis protein CobW	(Xiong et al., 2019)

		esterase, thioesterase, Polysaccharide deacetylase, ABC transporter, permease protein, ABC transporter, ATP binding protein, ABC transporter, sugar binding protein, GAF domain/GGDEF domain protein, ABC transporter, lipoprotein			
G.halophytocola DR408	Rhizospheric soil/ osmolytic stress	Trehalose-6-phosphate synthase, trehalose phosphatase, glutamate synthase, proline dehydrogenase, Na+/H+ antiporter and OsmC family protein		1-aminocyclopropane-1-carboxylate deaminase, tryptophan synthase, siderophore-interacting protein, iron-siderophore ABC transporter substrate-binding protein, glucose-6-phosphate dehydrogenase, phosphate ABC transporter permease	(Nishu et al., 2019)
G. mishrai S5–52^{T}	Coral *Favia veroni* / osmotic and cold stress	Superoxide dismutase, Catalase, Catalase (peroxidase I), Peroxiredoxin, Predicted redox protein, regulator of disulfide bond formation, Thioredoxin domain-containing protein, Thioredoxin reductase, Thiol-disulfide isomerase and thioredoxins, Predicted transcriptional regulators, Glycosyltransferase, 1,4-alpha-glucan branching enzyme, ADP-glucose pyro phosphorylase, Glucan phosphorylase, Type II secretory pathway, pullulanase PulA and related glycosidases, Trehalose-6-phosphate synthase, Trehalose-6-phosphatase, ABC-type proline/glycine betaine transport systems, ATPase components	NR	NR	(Das et al., 2020)

		ABC-type proline/glycine betaine transport systems, permease component<br><br>Periplasmic glycine betaine/choline-binding (lipo) protein of an ABC-type transport system (osmoprotectant binding protein),<br><br>Cold shock DNA binding domain,<br><br>Transcription elongation factor, Polyribonucleotide nucleotidyltransferase (polynucleotide phosphorylase), Ribosome-binding factor A<br><br>Translation initiation factor 1 (IF-1), Translation initiation factor 2 (IF-2; GTPase)			
Arthrobacter agilis strain L77	Sub-glacial Lake	putative cold shock, protein A, Molecular chaperone Hsp31, glyoxalase 3, Copper chaperone CopZ, Mercuric resistance operon regulatory protein	GDSL-like Lipase/Acylhydrolase, Lipase 1 precursor, GDSL-like Lipase/Acylhydrolase,<br><br>Lipase 1 precursor, GDSL-like Lipase/Acylhydrolase, Glucose-resistance amylase regulator, Trehalose synthase/amylase TreS, Alpha-amylase precursor, Alpha-amylase/pullulanase, Glucose-resistance amylase regulator, putative bifunctional chitinase/lysozyme precursor, Chitinase, Putative cysteine protease YraA, Flp pilus assembly protein, protease CpaA, Lon protease, Putative serine protease HtrA, Serine protease,	Phosphate transport system permease protein PstA, Phosphate import ATP-binding protein PstB, Phosphate transport system permease protein PstC, Alkaline phosphatase synthesis sensor protein PhoR, PhoH-like protein,	(Singh et al., 2016)

			CAAX amino terminal protease self- immunity,<br><br>CAAX amino terminal protease self- immunity,<br><br>Serine protease Do-like HtrA, Rhomboid protease GluP, ATP-dependent zinc metalloprotease FtsH, Putative ATP-dependent Clp protease ATP-binding subunit, CAAX amino terminal protease self- immunity, Minor extracellular protease vpr precursor, Flp pilus assembly protein, protease CpaA, CAAX amino terminal protease self- immunity, Putative serine protease HtrA, Putative metalloprotease, Putative zinc metalloproteasec/MT2700,<br><br>Modulator of FtsH protease HflK, ATP-dependent Clp protease ATP-binding subunit ClpX, ATP-dependent Clp protease proteolytic subunit 1, ATP-dependent Clp protease proteolytic subunit 2, ATP-dependent Clp protease adaptor protein ClpS, ATP-dependent zinc metalloprotease FtsH, Serine protease inhibitor-like protein, CAAX amino terminal protease self- immunity, Metalloprotease MmpA, Protease PrtS precursor, Protease 2, Protease synthase and sporulation, negative regulatory protein, Protease PrsW, Protease 3 precursor, β-galactosidase bgaB, β-galactosidase, β-galactosidase precursor		

Arthrobacter alpinus R3.8	Soil	cold shock proteins, antifreeze protein	Beta-hexosaminidase, N-acetylglucosamine kinase, N-acetylglucosamine-specific IIA component, N-acetylglucosamine-specific IIB component, N-acetylglucosamine-6-phosphate deacetylase, N-acetylglucosamine kinase, Beta-hexosaminidase, Chitinase, Predicted N-acetyl-glucosamine kinase 2, Glucosamine-6-phosphate deaminase, N-acetylglucosamine-6-phosphate deacetylase, salicylate 1-monooxygenase , imidazole glycerol phosphate synthase cyclase, enoyl-CoA hydratase, alkaline phosphatase, nitrilotriacetate monooxygenase, aliphatic amidase amiE, urease alpha subunit, urease gamma subunit	glutamine synthetase, glutamate synthase, nitrogen regulatory protein, ammonium transporter, monoamine oxidase, tryptophan synthase, anthranilate phosphoribosyltransferase, N-(5′-phosphoribosyl) anthranilate Isomerase, Trehalose phosphorylase, haloacid dehalogenase, Trehalose phosphate synthase, malto-oligosyltrehalose, trehalohydrolase, maltooligosyl trehalose synthase, spermidine synthase	(See-Too et al., 2017)
Arthrobacter sp. ERGS1-01	Glacier/ cold stress	cold-shock protein, ATP-dependent chaperone ClpB, molecular chaperone HtpG, glycine/betaine ABC transporter substrate-binding protein, glycine/betaine ABC transporter, sodium:proton antiporter, trehalose-phosphate synthase, trehalose synthase, peroxiredoxin, superoxide dismutase, catalase, universal stress protein UspA, phytoene synthase, 3-oxoacyl-ACP reductase, 3-oxoacyl-ACP synthase, UDP-N-acetylglucosamine 1-carboxyvinyltransferase, carbon starvation protein CstA, Glycogen debranching protein, ATP-dependent DNA helicase RecG, DNA mismatch repair protein MutT, ATP-dependent DNA helicase PcrA, Holliday junction	glucoamylase, alpha amylase, serine protease, pullulanase, lipase, alcohol dehydrogenase, alkaline phosphatase	NR	(Kumar et al., 2015)

		ATP-dependent DNA helicase RuvA, DNA helicase UvrD			
Arthrobacter alpinus ERGS4-06	Glacier/ cold stress	Cold-shock protein, glycine/betaine ABC transporter ATPase, glycine/betaine ABC transporter substrate-binding protein, glycine/betaine ABC transporter, sodium:proton antiporter, trehalose-6-phosphate synthase, peroxiredoxin, superoxide dismutase, universal stress protein UspA, phytoene synthase, 3-oxoacyl-ACP reductase, 3-oxoacyl-ACP synthase, UDP-N-acetylglucosamine 1-carboxyvinyltransferase, ATP-dependent DNA helicase RecG, DNA mismatch repair protein MutT, ATP-dependent DNA helicase PcrA, DNA helicase UvrD, recombinase RecA	alpha amylase, glucoamylase, esterase/lipase, serine protease, permease	NR	(Kumar et al., 2016)

NR- not reported
